# Neuro-immune interactions in tumors, immune-related disorders, and infections

**DOI:** 10.1038/s41392-026-02842-x

**Published:** 2026-10-07

**Authors:** Qing-Yuan Zhuang, Si-Wei Zhang, Zhi-Ming Shao, Yi Xiao, Yi-Zhou Jiang

**Affiliations:** 1 Key Laboratory of Breast Cancer in Shanghai, Department of Breast Surgery, Fudan University Shanghai Cancer Center, Shanghai, PR China; 2 Department of Oncology, Shanghai Medical College, Fudan University, Shanghai, PR China; 3 Shanghai Academy of Natural Sciences (SANS), Fudan University, Shanghai, China

**Keywords:** Neuroimmunology, Tumour immunology, Immunological disorders

## Abstract

The nervous and immune systems interact to form a complex network that sustains homeostasis and finely tunes immune responses. These interactions significantly influence the pathogenesis and progression of various diseases, such as tumors, immune-related disorders, and infections. Consequently, these interactions have become promising therapeutic targets. However, insufficient mechanistic research and the lack of candidate drugs have restricted the application of therapeutic strategies targeting neuro-immune interactions. In this review, we summarize three modes of neuro-immune interactions: local mechanisms, reflex-level organization, and central nervous system-wide regulation. Next, we elucidate the mechanisms of neuro-immune interactions across different diseases, discuss their variations, and highlight the spatiotemporal heterogeneity of these interactions. Additionally, understanding the dynamic crosstalk between specific neuronal and immune cell subtypes could reveal novel biomarkers and intervention points, facilitating more precise and personalized therapeutic strategies in diverse disease contexts. Furthermore, we dissect the current status of clinical trials and analyze the major challenges hindering effective clinical translation. Finally, we explore the potential of high-throughput sequencing technologies, such as single-cell omics and spatial omics, as well as neuro-electrophysiological methods, for in-depth investigation of related mechanisms. By integrating preclinical and clinical perspectives, this review aims to provide valuable insights into the therapeutic potential and future directions in the burgeoning field of neuro-immune interactions.

## Introduction

Given our understanding of the complex physiological interplay between the nervous and immune systems, emerging evidence underscores the critical role of neuro-immune interactions in systemic diseases, particularly tumors, immune-related disorders, and infections. Through highly coordinated crosstalk, the nervous and immune systems act as key regulators of physiological functions, sustaining homeostasis at both the local tissue and systemic levels. Together, they help preserve the physiological balance and defend the host against pathogens.^1–3^ Tumors, now widely regarded as systemic diseases, exhibit significant neuro-immune involvement.^4^ For instance, recent studies have revealed sensory and autonomic nerve-mediated regulation of the tumor microenvironment (TME) in pancreatic cancer, as well as circadian rhythm-dependent immune cell reprogramming that influences immunotherapy efficacy.^5,6^ Similarly, in immune-related disorders such as allergies, autoimmune diseases, and infections, peripheral nerves not only sense inflammatory signals but also actively modulate the local immune microenvironment through interactions with innate immune cells.^7–10^ As evidenced by recent studies, these mechanistic insights have spurred the development of novel therapies targeting neuro-immune interactions.^11–13^ Collectively, neuro-immune interactions are indispensable in systemic disease pathogenesis and present new opportunities for therapeutic intervention.

Emerging evidence has revealed conserved mechanisms of neuro-immune interactions across diverse systemic diseases. A paradigmatic example is the brain-spleen axis, the structural foundation of the cholinergic anti-inflammatory reflex.^14^ This axis not only suppresses lipopolysaccharide (LPS)-induced septic inflammation in mice but also facilitates the infiltration of myeloid-derived suppressor cells (MDSCs) into tumors, thereby impairing antitumor immunity.^15,16^ Moreover, it drives cardiac macrophage proliferation via sympathetic innervation and placental growth factor, facilitating cardiac adaptation in hypertensive stress.^17^ Additionally, through β1-adrenergic receptor (ADRB1) signaling, catecholamines induce CD8^+^ T-cell exhaustion in both chronic infections and tumors.^18^ These findings suggest that the mechanisms of neuro-immune interactions are evolutionarily conserved across disease states.

An imperative exists to develop a systematic, comparative framework elucidating neuro-immune interactions across diverse systemic diseases, including tumors, immune-related disorders, and infections, particularly in light of recent breakthroughs in basic and translational research. Over the past decade, a series of seminal reviews has shaped our understanding of these interactions. A landmark review published seven years ago integrated scattered observations, such as the innervation of immune organs and neurotransmitter-mediated modulation of immune cells, into a coherent framework, delineating local and long-range communication between the nervous and immune systems.^19^ Three years later, another influential review, reflecting on the biological essence of both systems, proposed intrinsic drivers and highlighted the critical question of “mapping immune information in the brain”.^20^ More recent reviews, driven by interest in brain-body interactions and interoception, have emphasized neuro-immune regulation across spatial (intracerebral, long-range, and peripheral) and temporal (development, migration, and function) dimensions.^21^ The concept of a neuro-immune connectome was proposed to systematically map neuron-immune interactions via omics technologies, providing an ambitious theoretical model.^22^ Other studies have focused on disease-specific contexts, neuronal subtypes, or distinct modes of interaction.^23–28^ Despite these advances, clinical translation remains limited, with few cross-disease comparisons or systematic overviews of the progress and challenges of clinical trials.

This review synthesizes contemporary insights into neuro-immune interactions, encompassing local mechanisms, reflex-level organization, and central nervous system (CNS)-wide regulatory roles. Drawing upon seminal studies, we assess the pathophysiological significance of neuro-immune interactions across diverse disease spectra. Additionally, we critically evaluate clinical trials targeting these pathways, highlight the major translational challenges, and propose actionable strategies for therapeutic optimization. By integrating these perspectives, this review aims to advance both conceptual and clinical progress in neuroimmunology.

## Modes of neuro-immune interactions

**Fig. 1:**
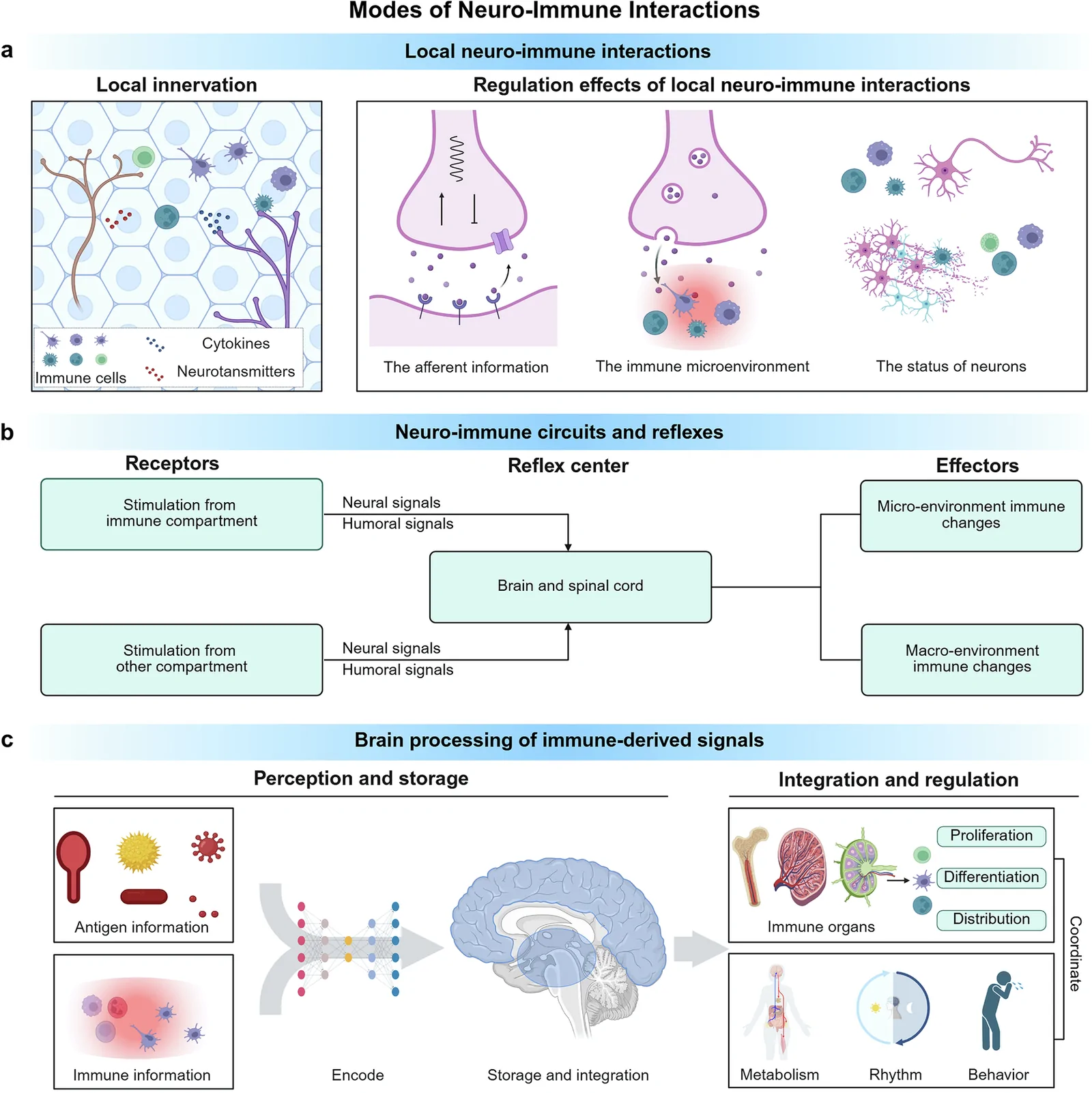
Modes of neuro-immune interactions. Neuro-immune interactions occur at three levels: local interactions (**a**), neuro-immune circuits and reflexes (**b**), and the processing of immune information in the brain (**c**). **a** In innervated tissues, neurons and immune cells communicate via cytokines, neurotransmitters, and other mediators, modulating neuronal input and state and shaping the immune microenvironment. **b** The nervous and immune systems form integrated reflex loops. The central nervous system detects immune signals through humoral and neural pathways, integrates this information, and regulates immune characteristics of both local and systemic environments via efferent outputs. **c** The brain senses, encodes, and interprets immune signals, coordinating immune responses, metabolism, and behavior to sustain homeostasis and respond to threats. The figure was created with BioRender.com

The nervous system employs two primary regulatory modalities: local regulation and reflex regulation, which represent distinct hierarchical control mechanisms. These processes furthermore involve CNS integration of sensory inputs.^29^ In this review, we introduce a conceptual framework for neuro-immune interactions, structured into three tiers, including local neuro-immune interactions, neuro-immune circuits and reflexes, and the processing of immune information in the brain (Fig. 1).

### Local neuro-immune interactions

Peripheral nerves exhibit dense anatomical integration within tissues and play a critical role in modulating local tissue homeostasis. Notably, neural infiltration has emerged as both a diagnostic and prognostic biomarker in various pathological conditions, particularly in cancer.^30–32^ Accumulating evidence reveals that local neuro-immune interactions form dynamic signaling networks between peripheral neurons and immune cells.^3,33,34^ Local interactions are characterized by bidirectional signaling that mediates critical biological functions, including the regulation of afferent information, the immune microenvironment, and the status of neurons.

The first key role of local neuro-immune interactions is the modulation of afferent signaling. Immune cells located near nerve endings can shape how sensory neurons perceive and transmit immune-related cues. Cytokines released by these immune cells lower the activation thresholds of sensory neurons, thereby increasing neural responsiveness during infection, inflammation, or allergy. For instance, during chronic allergic responses, γδ T-cell-derived interleukin-3 (IL-3) can enhance the activation of sensory neurons, driving the allergic immune response.^8^ Immune-mediated neuronal sensitization is particularly exemplified by mast cells, which express diverse receptors and secrete neuroactive mediators such as histamine shared with the nervous system.^35^ A study revealed that IgE-activated mast cells can produce protease chymase, activating transient receptor potential vanilloid 1 (TRPV1)^+^ sensory neurons by binding to protease-activated receptor-1 (PAR1).^36^ Moreover, evidence suggests that inflammation can increase afferent renal nerve activity.^37,38^ In addition, several studies have reported that immune cells can secrete neurotransmitters, such as gamma-aminobutyric acid (GABA) released by B cells and acetylcholine (ACh) produced by CD4⁺ T cells, B cells, and type 2 innate lymphoid cells (ILC2s).^39–42^ However, whether these neurotransmitters modulate afferent signaling in neurons has not yet been reported. In summary, molecules previously viewed as distinct from either the immune or nervous system have emerged as key signaling molecules that mediate communication between cells. This molecular overlap reflects deeper evolutionary convergence, as both systems are designed to sense, interpret, and adapt to environmental changes. Thus, the integration of immune and neural signaling is not incidental but rather biologically inevitable, given their shared logic of responsiveness and plasticity.

Second, local neuro-immune interactions play a regulatory role in immune responses. Peripheral neurons influence immune cell activity across diverse organs and tissues, including key immune structures such as the lymph nodes, spleen, and bone marrow, through direct cell-cell communications.^43–45^ Notably, neuronal innervation patterns, receptor expression profiles, and immune microenvironments exhibit significant organ-specific heterogeneity. Consequently, identical neural signals can induce divergent immune responses depending on the tissue context. Deciphering this complex interaction network remains a major challenge in the field. Sensory neurons secrete neuropeptides such as calcitonin gene-related peptide (CGRP) and substance P (SP), which modulate immune cell function.^46–48^ Neuron-derived SP, acting predominantly through the neurokinin-1 receptor (NK1R), enhances hematopoietic stem cell (HSC) proliferation in the bone marrow and primes peripheral monocytes for CCL5-dependent chemotaxis.^49^ In contrast, neuronal CGRP suppresses the macrophage proinflammatory phenotype by either directly interacting with intracellular NLRP3 or inhibiting downstream signaling pathways mediated by the RAMP1 receptor.^47,50–52^ The autonomic nervous system (ANS) influences immune responses via neurotransmitters, including norepinephrine (NE), epinephrine, and ACh.^53^ Adrenergic neurons regulate immune cells primarily through noradrenergic pathways. Adrenergic receptors, particularly ADRB2, are expressed on diverse immune cells, including myeloid cells, T and B lymphocytes, natural killer (NK) cells, and ILCs.^54–57^ Neuronal NE release enhances the anti-inflammatory program of macrophages in murine chemical peritonitis.^58^ Additionally, recent studies suggest that loss of adrenergic activity induces interferon-γ (IFN-γ) expression in CD8^+^ T cells.^59^ Cholinergic neurons primarily utilize ACh. The immunoregulatory effects of ACh are complex and depend on the specific receptors involved. For instance, cholinergic signaling through the ACh receptor M1 (CHRM1) reduces CD11b⁺ myeloid cells in pancreatic ductal adenocarcinoma (PDAC), whereas signaling through CHRM3 promotes CD4⁺ T-cell activation and cytokine production during helminth and bacterial infections.^60,61^ These findings collectively demonstrate that neuro-immune communications constitute a sophisticated, multilayered network rather than simple linear interactions.

Third, immune cells directly influence neuronal states through local interactions. These cells modulate neuronal phenotypes by regulating key cellular processes, including proliferation and apoptosis. Neuronal sprouting, growth, and infiltration are regulated by neurotrophins and cytokines secreted by immune cells, including mast cells and macrophages.^62,63^ The gut represents a unique site of immune-neural crosstalk. In the gut, intestinal resident macrophages refine the ENS by pruning synapses and phagocytosing enteric neurons.^64^ The latest research also revealed that microglia are present in the peripheral nervous system (PNS), and they directly envelop neuronal cell bodies, forming a “neuron-microglia-satellite glial cell” triad structure and effectively promoting axonal growth and the somatic enlargement of peripheral neurons.^65^ This study demonstrates the conservation of neuro-immune interactions across nervous system compartments. Collectively, the immune system critically shapes neuronal states, remodeling the neural landscape both structurally and functionally to sustain tissue homeostasis.

### Neuro-immune circuits and reflexes

Reflexes represent a fundamental neurophysiological mechanism that mediates rapid adaptive responses to environmental stimuli. Both conditioned and unconditioned reflex arcs facilitate survival by optimizing beneficial responses while mitigating potential harm. Innate reflexes, such as the baroreflex, sneezing reflex, and respiratory reflex, are crucial for sustaining physiological homeostasis.^66–69^ The discovery of anti-inflammatory reflexes dates back several decades, with early clinical applications explored shortly thereafter. Recent studies have elucidated the intricate mechanisms governing these reflexes.^70–72^ These findings indicate that neural reflexes exert regulatory control over the immune system. Neuro-immune reflexes possess the fundamental components of a reflex, including effectors, receptors, and reflex centers.^73^ Previous studies have reported that the nuclei in the brainstem and mesenteric ganglion serve as reflex centers in neuro-immune reflexes.^74,75^ In classical reflex theory, afferent and efferent neural pathways are defined as parts of neural reflexes.^73^ Here, we accept the concept of neurohumoral regulation to include hybrid transmission of neural and humoral signals, given that immune cells primarily communicate through humoral mechanisms.^76,77^ In contrast to traditional neural reflexes that elicit transient responses, neuro-immune reflexes demonstrate sustained adaptive properties due to immune system involvement. For instance, repeated allergen exposure induces persistent behavioral avoidance alongside immune activation, including mast cell degranulation.^78–80^ This underscores the profound functional synergy and complementarity between the immune and nervous systems. A prior review conceptualized the nervous system from an immunological standpoint as the “behavioral immune system”, reflecting its role in the rapid immune response.^81^

Recent research has significantly advanced our understanding of neuro-immune reflexes. The cholinergic anti-inflammatory reflex represents a well-defined neuro-immune reflex. This reflex involves a multistep axis: vagus nerve activation stimulates splenic adrenergic nerves, which signal to ADRB2-expressing T cells, ultimately modulating innate immune cells.^71^ Specifically, vagal stimulation activates splenic adrenergic nerves that relay signals to ADRB2^+^ T cells. These T cells synthesize ACh, inhibiting myeloid cell activity via the α7 nicotinic ACh receptor (α7nAChR) pathway. Additional studies have confirmed ADRB2 expression in T cells and ACh production by choline O-acetyltransferase (ChAT)^+^ T cells.^82–84^ Consequently, this reflex may represent a conserved mechanism of neuro-immune crosstalk in innervated tissues. The mesenteric ganglion has been identified as a critical regulatory hub in neuro-immune reflexes, where it exerts immunosuppressive effects on mesenteric lymph nodes and the spleen, thereby expanding the known functional scope of the cholinergic anti-inflammatory reflex.^75^ Together, these findings demonstrate that neural reflex arcs function as precise regulatory mechanisms in disease pathogenesis, shaping immune responses both locally and systemically.

### Brain processing of immune-derived signals

Emerging evidence has demonstrated that the brain actively monitors, encodes, and modulates immune responses, forming a bidirectional communication network between neural circuits, peripheral immunity, and systemic physiology. A recent study employed activity-dependent cell labeling in mice to identify insular cortex (InsCtx) neurons activated during colitis.^85^ Reactivating these neurons alone was sufficient to reproduce the inflammatory state, demonstrating that the brain encodes and retrieves specific immune responses. This work expands the classical concept of immunological memory, previously attributed to clonal memory lymphocytes or the epigenetic alterations of these immune cells.^86,87^ Another study identified a neural pathway linking corticotropin-releasing hormone (CRH) neurons in the amygdala and paraventricular nucleus (PVN) to the spleen.^72^ This pathway selectively enhances vaccine-induced antibody responses and is modulated by behavioral cues. These findings indicate that the brain not only elicits targeted immune responses but also reprograms peripheral immunity. The brain integrates signals across regions governing distinct physiological systems, establishing multisystem regulatory networks. For instance, adipocytes signal to the CNS via afferent neurons to adjust pancreatic insulin secretion.^88^ Similarly, a liver-brain-gut neuro-immune reflex has been shown to sustain intestinal regulatory T (Treg) cell homeostasis.^89^ Thus, the brain exerts hierarchical regulation over whole-body physiology. Mechanistic studies dissecting brain-neural circuit-immune interactions could yield insights into systemic pathologies, including cancer immunobiology.

Building upon this framework, we systematically examine the conserved mechanisms and disease-specific adaptations of neuro-immune interactions across systemic pathological contexts, including tumors, immune-related disorders, and infections.

## Neuro-immune interactions in tumors

**Fig. 2:**
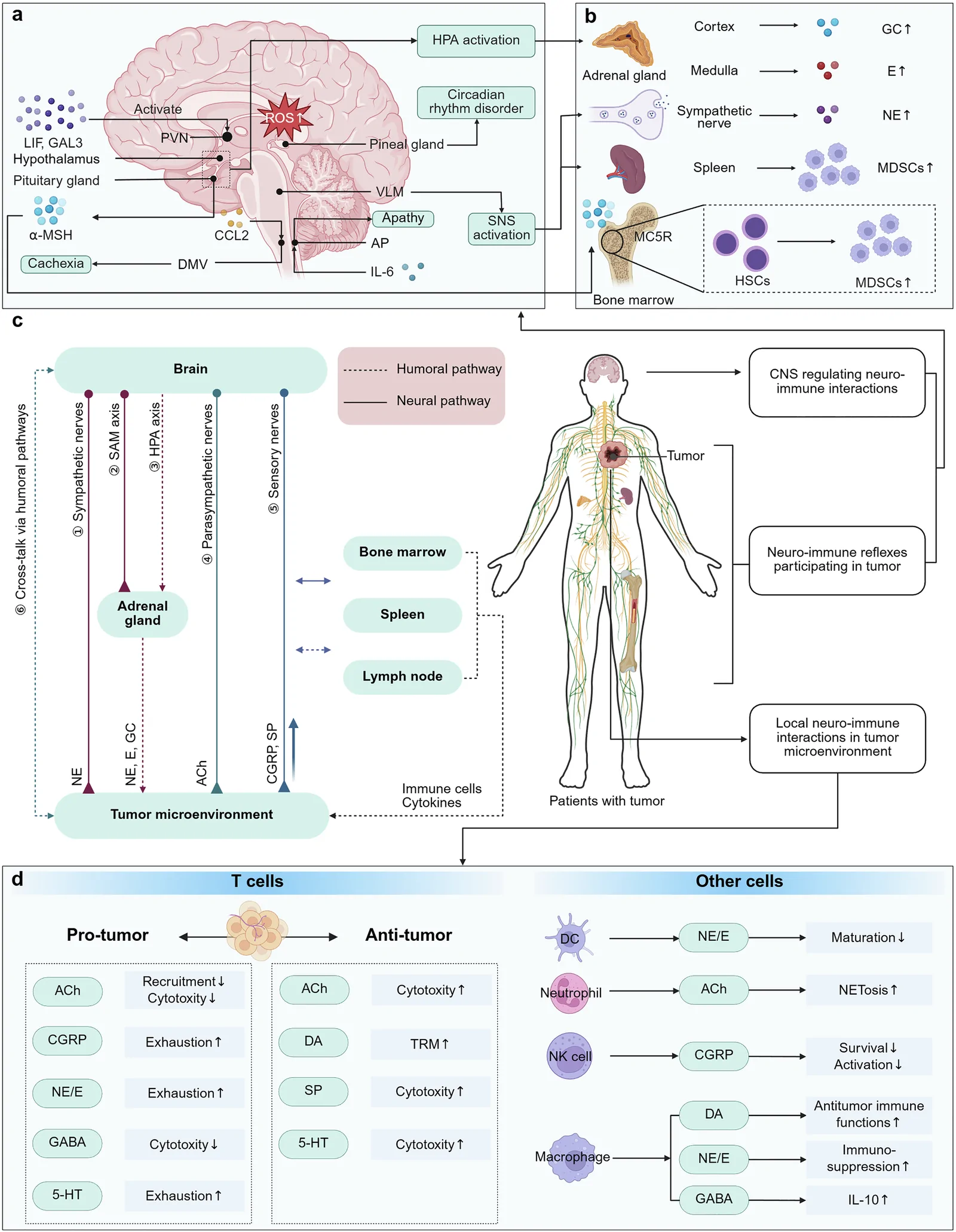
Neuro-immune interactions in tumors. Neuro-immune interactions in tumors encompass three levels (**c**): the CNS (**a**), reflex circuits (**a**, **b**), and the TME (**d**). **a** Tumor-derived signals, such as secreted proteins, are sensed and integrated by the CNS, leading to activation of the SNS and HPA axis and contributing to neuropsychiatric symptoms and cachexia progression. **b** Tumors can induce immunosuppression by hijacking the HPA axis, the brain-spleen axis, and the pituitary-bone marrow axis. The upper left portion illustrates brain regions activated in the tumor context. **c** Interactions between the nervous system and the TME occur via neural and neurohumoral pathways, integrating signals from the CNS and reflex circuits. On the left side, solid lines represent the neural pathway, while dashed lines represent the humoral pathway. In the neural pathway, red, green, and blue indicate sympathetic, parasympathetic, and sensory nerves, respectively. The inverted triangles at the ends of the lines represent nerve fibers projecting into the TME. **d** Immune cells in the TME are modulated by neurotransmitters and neuropeptides through corresponding receptors. The figure was created with BioRender.com. ACh acetylcholine, α-MSH alpha-melanocyte-stimulating hormone, AP area postrema, CCL2 C-C motif chemokine ligand 2, CGRP calcitonin gene-related peptide, DA dopamine, DMV dorsal motor nucleus of the vagus, E epinephrine, GAL3 galectin-3, GABA gamma-aminobutyric acid, GC glucocorticoid, HPA hypothalamic-pituitary-adrenal, LIF leukemia inhibitory factor, MDSCs myeloid-derived suppressor cells, NE norepinephrine, NP neuropeptide, 5-HT 5-hydroxytryptamine, PVN paraventricular nucleus, TRM tissue-resident memory T, VLM ventrolateral medulla

Emerging evidence indicates that tumor-mediated immune regulation has surpassed the traditional concept of local tissue boundaries, encompassing complex reprogramming of the systemic macroenvironment.^90^ Within this process, the nervous system has emerged as a central integrative hub linking the local TME to the systemic macroenvironment and is increasingly recognized as a new hallmark of cancer.^91^ Peripheral nerves penetrate tumor tissues through dense neural fiber networks, releasing neurotransmitters and neuropeptides influencing tumor cell proliferation, metastasis, and immune cell activity.^4,92^ At the central level, the bidirectional regulatory network formed by the hypothalamic-pituitary-adrenal (HPA) axis and the sympathetic nervous system (SNS) is of particular importance, participating in establishing a systemically immunosuppressive macroenvironment.^83,93,94^ This profound tridirectional interplay among nerves, immune cells, and tumors highlights the complexity of cellular interactions within the TME (Fig. 2).

### Local neuro-immune interactions in tumors

Neurons have already been recognized as components of the TME and are associated with tumor growth, invasion, metastasis, and therapeutic resistance, especially in tumors with a high incidence of perineural invasion (PNI), including pancreatic cancer.^31,95–101^ However, due to nerves infiltrating tumors mainly as axons and the limited availability of representative tissue samples, the role of nerves in the TME remains underexplored. Emerging findings have redefined neurons in the TME from passive “bystanders” to active “participants” in shaping the tumor immune ecology.^102–104^ Local neuro-immune interactions within the tumor are not limited to direct neuronal innervation. Neurotransmitters can be secreted by cancer cells and immune cells in the TME, and these neurotransmitters can regulate immune responses through neuron-independent mechanisms.^39,105^ Furthermore, tumors exhibit plasticity. Recent studies have revealed that tumors can mimic immune mechanisms within the nervous system to achieve immune escape, offering a new perspective on local neuro-immune interactions.^106^ In this section, we summarize recent findings on local neuro-immune interactions in three contexts, including infiltration of peripheral neuronal subtypes, other neurotransmitter-mediated regulation, and the neuro-mimetic phenotype of tumors.

#### Sensory nerves

Sensory nerves constitute a critical component of the PNS, capable of shaping the local immune microenvironment through the secretion of neuropeptides and playing a pivotal role in cancer pain pathogenesis. Among these, SP and CGRP are two significant neuropeptides released by sensory neurons that play a dual role in antitumor immunity. Recent studies have demonstrated that SP, secreted by sensory neurons, binds to TACR1-expressing dying cancer cells, triggering the release of single-stranded RNA (ssRNA). This ssRNA subsequently activates Toll-like receptor 7 (TLR7) in neighboring tumor cells, inducing a pro-metastatic gene expression program.^100^ Although the TLR family primarily functions in pathogen sensing and immune cell proliferation, this study highlights the potential for indirect immune modulation by sensory nerves. Contrasting evidence suggests that SP may enhance antitumor immunity. For instance, SP increases the population of CD4^+^CD25^bright^ cells in tumor-draining lymph nodes and elevates IFN-γ secretion in leukocyte cultures from tumor-bearing mice while reducing MDSC infiltration, thereby promoting cytotoxic immunity in the presence of radiotherapy and preventing metastatic growth.^107^ CGRP also plays an important role in the TME. In a gastric cancer mouse model, a dense distribution of CGRP^+^ sensory neurons was observed in tumor tissues. This dense distribution was attributed to the high expression of nerve growth factor (NGF) by gastric cancer cells.^108^ These neurons release CGRP, which promotes the proliferation of platelet-derived growth factor receptor alpha (PDGFRA)^+^ tumor-associated fibroblasts and shapes the tumor immune microenvironment. Recent studies on PDAC have identified a critical role for the sensory neuron-cancer-associated fibroblast (CAF)-tumor immune microenvironment axis.^5^ CGRP, released by nociceptive neurons, binds to receptor activity-modifying protein 1 (RAMP1)/calcitonin receptor-like receptor (CALCRL) on CAFs, thereby suppressing IL-15 secretion. IL-15 is essential for NK cell survival and activation; its downregulation results in impaired NK cell infiltration and functional exhaustion, ultimately promoting tumor progression.^109^ Notably, the oral CGRP receptor antagonist rimegepant was found to significantly suppress PDAC growth, prolong survival time, and alleviate pain.^5^ In melanoma, a separate study elucidated the mechanism underlying CGRP release by sensory neurons. Melanoma-derived secretory leukocyte peptidase inhibitor (SLPI) acts on TRPV1^+^ nociceptive neurons, upregulating Calca (calcitonin-related polypeptide alpha, the gene encoding CGRP) and enhancing CGRP release. The CGRP-RAMP1 signaling axis drives CD8^+^ T-cell exhaustion, facilitating melanoma progression.^110^ Furthermore, co-culture of melanoma cells with nociceptive neurons upregulated the expression of Trka (tropomyosin-related kinase A), a gene implicated in neuronal sensitization. Collectively, these mechanisms illustrate how neurons modulate the immune system and central perception in response to cancer-derived pain signals. A study comparing the single-cell landscapes of medullary thyroid carcinoma and papillary thyroid carcinoma revealed that CGRP expression in medullary thyroid carcinoma was associated with aberrant DC development.^111^ This aberrant development was characterized by the activation of the cAMP-related pathway and elevated levels of Kruppel-like factor 2 (KLF2), which correlated with impaired activity of tumor-infiltrating T cells. A recent study took a novel approach by leveraging the pseudounipolar structure of sensory neurons, combined with neural retrograde tracing and sensory neuron-specific chemogenetic and optogenetic mouse models.^112^ Researchers have reported that the activation of sensory nerves innervating the tumor could further activate interconnected sensory neurons innervating the tumor-draining lymph nodes. These activated neurons subsequently release CGRP, which suppresses antitumor immune responses within the lymph nodes. In summary, existing evidence indicates that CGRP directly or indirectly restrains the function of antitumor immune cells, such as CD8⁺ T cells and NK cells.

#### Autonomic nerves

The ANS critically shapes the immunological properties of tumors. Both the sympathetic and parasympathetic nerves, which regulate organ homeostasis, infiltrate and innervate various solid organs.^113–115^ In tumors across diverse organs, autonomic nerve fiber infiltration has been documented to modulate the TME.^97,116^

Sympathetic nerves modulate T-cell function within the TME. In PDAC, NE released by sympathetic nerves suppresses CD8^+^ T-cell antitumor responses via ADRB1.^18^ In immune checkpoint blockade (ICB)-resistant PDAC mouse models, combined β-adrenergic blockade and ICB therapy augmented CD8^+^ T-cell responses and fostered the formation of tissue-resident memory-like T cells. Elevated immune checkpoint expression is a hallmark of T-cell exhaustion.^117,118^ Propranolol, a β-adrenergic receptor antagonist, diminishes programmed death-1 (PD-1) expression on CD8^+^ T cells in melanoma mouse models. Meanwhile, ADRB2 knockout produces similar effects.^119,120^ Additionally, the influence of the SNS on the TME is pleiotropic, extending beyond the direct regulation of T-cell function. Studies in breast cancer, colorectal cancer, melanoma, and immunologically “cold” head and neck tumors separately indicate that sympathetic signaling promotes an immunosuppressive phenotype in macrophages and impairs DC maturation, thereby suppressing adaptive immunity and fostering an immunosuppressive TME.^99,121–123^ Another study utilizing mouse models of breast cancer and lymphoma revealed that ADRB2 activates the immunosuppressive function of MDSCs by upregulating oxidative phosphorylation and fatty acid oxidation while suppressing glycolysis.^124^ Multiple in vitro studies have confirmed that sympathetic neurotransmitters such as NE and epinephrine can induce tumor cells to secrete various cytokines, including IL-6 and IL-8.^125,126^ These observations suggest that the SNS might modulate the immune microenvironment via mechanisms beyond T-cell-mediated pathways. Owing to its broad distribution, the SNS can modulate the microenvironment in both tumor and non-tumor tissues. For example, recent studies have demonstrated that sympathetic nerves contribute to premetastatic niche formation.^127^ Sympathetic activation upregulates chemokine (C-C motif) ligand 2 (CCL2) in lung stromal cells and C-C chemokine receptor type 2 (CCR2) in monocytes and macrophages, thereby promoting macrophage recruitment to premetastatic lungs. Chronic stress, a potent SNS activator, enhances neutrophil infiltration into the lungs and ACh-mediated neutrophil extracellular trap (NET) formation, a process linked to dormant tumor cell reactivation and metastatic progression.^128,129^ Beyond tumor sites, sympathetic nerves innervate lymphoid organs such as lymph nodes, spleen, and bone marrow to regulate immune cell mobilization during stress responses and homeostasis.^44,130^ Under homeostasis, sympathetic signaling governs lymph node trafficking rhythms, HSC egress from bone marrow, and vascular-mediated immune cell recruitment into tissues.^131^ This warrants further investigation of how such sympathetic innervation changes within tumors and how these alterations influence the tumor itself.

Previous studies have indicated that intratumoral infiltration of sympathetic nerves has tumor-promoting immunomodulatory effects.^132,133^ However, surprisingly, research related to pancreatic cancer has revealed the tumor-suppressing role of intratumoral sympathetic nerve activation. During pancreatic cancer progression, sympathetic nerve density progressively decreases.^134^ Animal models have demonstrated that surgical sympathectomy accelerates pancreatic cancer progression and is associated with increased infiltration of CD163^+^ macrophages within the TME following sympathetic denervation.^135^ Accordingly, preserving sympathetic nerve innervation exerts a protective effect against pancreatic cancer. However, studies in pancreatic cancer have revealed that NE suppresses antitumor activity via ADRB1⁺ T cells.^18^ Additional murine studies have shown that peripheral sympathectomy downregulates natural killer group 2D (NKG2D) and CCR5 expression in NK cells, impairing their antitumor activity in an enriched environment.^136^ This paradox highlights the complexity of neuro-immune interactions in cancer. In summary, categorizing sympathetic nerves simply as “pro-tumorigenic” or “anti-tumorigenic” is no longer adequate to capture the complexity of tumor biology. To address this, we propose a dynamic model in which the outcomes of neuro-immune regulation are jointly determined by disease stage, receptor-specific engagement, and the local decoupling of neurotransmitter sources from their targets. The core assumptions of this model include the following: (1) the effects of autonomic nerves (such as the SNS) on tumor immunity are both dose- and time-dependent; and (2) the ultimate outcome is shaped by the receptor subtype expression profile and specific functions within different tissue contexts. The first assumption is consistent with the physiological principle that sympathetic activity regulates blood flow in a dose- and time-dependent manner.^137,138^ Evidence supports the temporally dynamic features of neuro-immune modulation. Acute sympathetic activation rapidly increases the counts of neutrophils, eosinophils, and NK cells while also promoting lymphocyte mobilization, thereby enhancing anti-infective immunity.^139–141^ In contrast, chronic sympathetic activation, such as during persistent stress, tends to exert anti-inflammatory effects and inhibit immune responses.^142,143^ The second assumption reflects the heterogeneity of innervation patterns and receptor-ligand interactions across tissues. Different tumors and other tissues exhibit substantial differences in stromal composition, immune infiltration patterns, and innervation density. These variations allow identical neurotransmitters to induce opposing biological effects via differential receptor subtype engagement or downstream signaling cascades, similar to the effect of the sympathetic nerves on the heart.^144^ A representative example is that hypothalamic brain-derived neurotrophic factor (BDNF) downregulates leptin production in adipocytes via sympathetic β-adrenergic signaling, thereby delaying the progression of melanoma and colorectal cancer in mice.^145^ In this context, the SNS exerts a tumor-protective effect by modulating the systemic macroenvironment. Another study further indicated that the origins of tumor cells influence neuro-tumor interactions. By maintaining the bone marrow hematopoietic niche, sympathetic innervation can instead confer a protective effect against acute myelogenous leukemia development.^146^ Furthermore, a potential “source-target decoupling” phenomenon in neurotransmitter action may occur, as previously reported in a seminal study on depression.^147^ Although pancreatic cancer exhibits reduced sympathetic innervation density^134^, local NE levels may be sustained through alternative mechanisms, including paracrine synthesis by TME cells and circulatory leakage. For example, recent studies have reported that T cells within the tumor microenvironment can synthesize another monoamine neurotransmitter, 5-HT, and activate their own functions through an autocrine mechanism, a pathway that is independent of plasma-derived 5-HT.^148^ Whether a similar mechanism operates for NE remains to be determined.

The parasympathetic nervous system (PaNS) mediates immunoregulation primarily via ACh signaling through muscarinic and nicotinic receptors. In multiple solid tumors, parasympathetic innervation originates from vagal nerve projections.^60,149^ Research in murine lung cancer models has demonstrated that vagal nerve stimulation (VNS) alone significantly enhanced the activation profile of intratumoral CD8^+^ T cells, as evidenced by the upregulation of IFN-γ and CD137 expression.^150^ However, ACh impedes CD8^+^ T-cell recruitment via histone deacetylase 1 (HDAC1)-mediated CCL5 suppression in PDAC cells.^151^ Furthermore, ACh dose-dependently suppresses IFN-γ production by CD8^+^ T cells while promoting T helper 2 (Th2) over Th1 polarization.^151^ Similar immunosuppressive effects are observed in colorectal and prostate cancers. The expression of CHRM3 and CHRM4 positively correlates with the expression of immunosuppressive markers within the TME.^152,153^ The existing research also presents inconsistent conclusions. PaNS modulates myeloid cell activity. In pancreatic cancer models, enhanced cholinergic signaling suppresses CD11b^+^ myeloid cells and reduces TNF-α levels, thereby promoting antitumor immunity.^60^ These findings suggest that normal vagal tone may help maintain antitumor immunity and indicate that the relationship between parasympathetic activity and the tumor immune phenotype may not be linear. Notably, tumors are capable of synthesizing ACh. A study on pancreatic cancer demonstrated that cholinesterase is highly expressed in human pancreatic tumor tissue samples.^154^ In vitro experiments revealed that either cholinesterase inhibitors or direct application of ACh agonists could suppress pancreatic cancer cell proliferation and invasion and reduce TAM infiltration by inhibiting the pERK signaling pathway. This mechanism is consistent with the assumptions we made when discussing sympathetic nerves; it represents “source–target decoupling,” acting as a confounding factor in describing the relationship between cholinergic neural activity and antitumor immunity.

Together, these findings further underscore the high complexity of neuro-immune regulation in cancer. First, the sources of regulatory signals are diverse and not limited to neural origins; second, the composition of the immune microenvironment varies significantly across tumors of different tissue types, disease stages, and molecular characteristics, suggesting that neural signals may exert distinct effects on different cell types.

#### The impact of other neurotransmitters on local tumor immune characteristics

In addition to autonomic and sensory nerves, both of which modulate immune cells through neurotransmitter release, various other neurotransmitters also exhibit immunomodulatory properties. These neurotransmitters are not exclusively neuron-derived; immune cells and tumor cells within the TME can synthesize specific neurotransmitters, directly shaping immune phenotypes.^155–157^

GABA, the primary inhibitory neurotransmitter in the CNS, has been found to play a crucial role in promoting immunosuppressive phenotypes across multiple cancers, including lung and colorectal malignancies.^105,158^ Tumor cells with aberrant expression of glutamate decarboxylase 1 (GAD1) synthesize GABA. Tumor-derived GABA subsequently activates GABA_B_ receptors, leading to inhibition of GSK-3β and enhancement of β-catenin signaling, which promotes tumor proliferation and limits CD8⁺ T-cell infiltration.^105^ Similarly, tumor-associated B cells secrete GABA, promoting monocyte differentiation into IL-10-producing macrophages and impairing CD8^+^ T-cell-mediated antitumor responses.^39^ In lung adenocarcinoma, GABA inhibits nuclear factor-κB (NF-κB) and signal transducer and activator of transcription 3 (STAT3) signaling pathways, thereby suppressing the antitumor phenotype of macrophages.^159^ Concurrently, GABA promotes the differentiation of macrophages toward an anti-inflammatory phenotype via activation of the STAT6 signaling pathway.^160^ Furthermore, GABA upregulates the expression of fibroblast growth factor 2 (FGF2) in macrophages, which subsequently enhances neovascularization within the tumor tissue.^159^ Preclinical studies in breast cancer models have demonstrated that GABA and its derivative baclofen upregulate immune checkpoint programmed cell death ligand 1 (PD-L1), which in turn can enhance the response to immunotherapy.^161^

Dopamine, a key neurotransmitter regulating emotion and reward, also modulates antitumor immunity. Dopamine primarily exerts its regulatory effects through dopamine receptors. Dopamine receptors are classified into D1-like and D2-like subtypes. The D1-like group includes the D1 and D5 receptors, which, upon activation, stimulate cAMP production via Gs proteins. In contrast, the D2-like group comprises the D2, D3, and D4 receptors, which reduce cAMP levels through Gi proteins.^162^ In murine colorectal cancer models, dopamine signaling via D5 promotes CD8^+^ T-cell differentiation into tissue-resident memory subsets, enhancing their antitumor activity and suppressing tumor growth.^163^ With respect to D2-like receptors, in breast cancer, the D2 receptor reprograms TAMs into a phenotype characterized by antitumor immune functions while simultaneously triggering GSDME-mediated pyroptosis.^164^ In summary, dopamine acts as a double-edged sword in tumor immunity: depending on the receptor subtype and concentration, it can either enhance or suppress T-cell antitumor responses.

5-HT, another emotion-linked neurotransmitter, exerts systemic effects via widely distributed receptors, modulating gastrointestinal and vascular physiology.^165–167^ In mice, deficiency of peripheral 5-HT, which mainly originates from the gut, reduces tumor cell PD-L1 expression through transglutaminase 2 (TGM2)-mediated serotonylation, thereby increasing functional CD8^+^ T-cell accumulation.^168^ High-dose selective serotonin reuptake inhibitors (SSRIs) (20 mg/kg) potentiate the efficacy of PD-1 checkpoint blockade in preclinical models, enabling durable tumor control through reducing peripheral 5-HT.^168^ Another study revealed an alternative mechanism by which 5-HT activates T cells. When serotonin transporter (SERT)-mediated reuptake is inhibited, the concentration of 5-HT outside T cells increases, and T-cell-autonomous 5-HT signaling via 5-HTR2B/7 receptors augments CD8⁺ T-cell effector functions.^148^ This study also proposes the use of SSRIs (10 mg/kg), a class of antidepressants, to enhance antitumor immunity by modulating local serotonin availability. This mechanism of 5-HT uptake via SERT actually resembles the distribution of neurotransmitters between neural synapses. Interestingly, the accumulation of intracellular 5-HT in T cells is also of potential value. The uptake of 5-HT and TGM2-mediated serotonylation of GAPDH promotes glycolysis in CD8⁺ T cells, thereby enhancing antitumor immunity.^169^ These studies appear to yield divergent conclusions regarding the roles of 5-HT in CD8^+^ T-cell function, yet these findings are not contradictory. The first study highlights the critical role of programmed cell death ligand 1 (PD-L1), demonstrating that 5-HT promotes tumor PD-L1 expression, thereby inducing T-cell exhaustion, while high-dose SSRIs are used to deplete peripheral 5-HT. The second study emphasizes the function of membrane 5-HT receptors on T cells, indicating that T cells can store and release 5-HT autonomously. By limiting reuptake, intracellular 5-HT is translocated extracellularly to activate membrane receptors, thereby enhancing CD8^+^ T-cell function. Here, SSRIs are employed to inhibit reuptake. The final study underscores the regulation of metabolism through the serotonylation of metabolic enzymes, emphasizing that intracellular accumulation of 5-HT modulates metabolic pathways and enhances T-cell function.

#### Neuro-mimetic phenotype of the tumor

Phenotypic plasticity has been increasingly recognized as a hallmark of cancer. Trans-differentiation may contribute to this process, in which cells originally committed to a specific differentiation pathway switch to an entirely distinct developmental program, thereby acquiring tissue-specific phenotypes not present in their cells of origin.^170^ An increasing body of evidence indicates that diverse malignancies, such as breast and pancreatic cancers, can acquire a neuro-mimetic phenotype through epigenetic remodeling and transcriptional reprogramming, thereby facilitating tumor progression.^171,172^ By mimicking features of neuro-immune interactions under physiological conditions, this neuro-mimetic phenotype may shape an immunosuppressive microenvironment conducive to tumor survival and invasion.

Specifically, in breast cancer, tumor cells can express the neuro-metabolic enzyme NAT8L and produce its metabolite N-acetylaspartate (NAA), thereby inhibiting immune synapse formation in NK cells and cytotoxic T cells. This mechanism suggests that breast cancer may achieve immune evasion by mimicking anti-inflammatory strategies used by the CNS.^106^ Another study reported the presence of a conserved population of “interface” tumor cells at the boundary between melanoma and normal tissue. Under mechanical stress, these cells express neural-like transcriptional programs (involving the upregulation of genes such as NNAT, NEFM, and SOX11) and exhibit increased invasive capacity and drug resistance. Although the study did not thoroughly investigate the direct interaction between these cells and the immune microenvironment, their location at the tumor margin, where immune responses are typically active, suggests that they may participate in local immunosuppression.^78^

As an emerging research focus, neuro-mimicry presents several key scientific questions that warrant further investigation. One important question is how tumors emulate the “immune privilege” characteristic of the nervous system through neuro-mimetic mechanisms, a phenomenon of substantial biological significance. Second, whether neuro-mimetic tumor cells can actively sculpt an immune-rejecting or suppressive microenvironment, analogous to that of neural tissues, through the secretion of neural-related metabolites or the expression of neural-specific proteins is another critical direction for future research. Addressing these questions will not only deepen the understanding of tumor immune evasion mechanisms but may also provide a theoretical foundation for developing novel therapeutic strategies that target the interaction between neuro-mimicry and the tumor immune microenvironment.

### Neuro-immune reflexes in the tumor

The neuro-immune reflexes constitute a critical regulatory axis within tumor immunity. The neuro-immune reflexes, which connect interoception with homeostatic maintenance mechanisms, play a significant role in brain-body crosstalk.^29^ Within the tumor context, a tumor possesses a profound capacity to shape its macroenvironment, including the immune and metabolic landscapes.^90^ Harnessing the neuro-immune reflexes to reprogram the tumor immune microenvironment and even cross-organ immune homeostasis may represent a pivotal pathway through which tumors acquire immune characteristics favorable for their own survival.^173^

The vagus nerve-mediated anti-inflammatory reflex is the most extensively studied neuro-immune reflex in oncology. While it systemically curbs inflammation in cancer patients, such as by reducing the levels of cytokines, including IL-6, IL-1β, and TNF-α, it simultaneously enhances the intratumoral recruitment of immunosuppressive cells such as MDSCs, thereby impairing antitumor immunity.^174^ The tumor-secreted proteins leukemia inhibitory factor (LIF) and galectin-3 (GAL3) activate the PVN to induce splenic nerve excitation, which in fact enhances MDSC production and intratumoral infiltration through the anti-inflammatory reflex pathway.^16^ Vagally activated memory T cells secrete trefoil factor 2 (TFF2) in the spleen, which suppresses MDSC proliferation via CXCR4 signaling, consequently inhibiting colorectal cancer development, similar to a mechanism that complements the inflammatory reflex.^175^ Encouragingly, both clinical applications and preclinical models of VNS have shown potential benefits, underscoring the therapeutic promise of targeting neural pathways to modulate systemic inflammation.^150,176,177^ Nevertheless, the role of the anti-inflammatory reflex in oncology remains insufficiently explored. Existing studies have focused primarily on its downstream effects, particularly those centered on the spleen as a key effector organ. In contrast, the afferent limb of this reflex, specifically how tumors initiate signaling and whether they can actively hijack this reflex, remains poorly understood.

Neuro-immune reflexes may also be mediated through alternative mechanisms. For instance, chronic stress promotes cellular senescence and impairs the stemness of intestinal stem cells via activation of the parasympathetic pathway in the central amygdala-dorsal motor nucleus of the vagus (CeA-DMV) circuit.^178^ The results imply that tumors could modulate gut homeostasis via the ANS pathway, which may consequently alter the immune macroenvironment due to the critical role of the gut in the immune system.^179^ A recent study provided clues regarding the involvement of the liver. Systemic inflammation induced by cancer can alter vagal tone, specifically the afferent pathway of the vagus nerve-mediated reflex. This vagal dysfunction disrupts the brain-liver-vagal axis, leading to depletion of HNF4α, which reprograms hepatic protein metabolism, exacerbates systemic inflammation, and promotes the development of a cachexia phenotype.^180^ Tumors modulate autonomic nerve activity by hijacking neuro-immune reflex pathways, which may indirectly participate in the regulation of immune cells by the ANS.

In addition to unconditioned reflexes such as the inflammatory reflex, studies have also demonstrated the involvement of conditioned reflexes in the immune system.^181^ Although research on immune conditioning in the field of oncology remains limited, immune conditioning may hold significant therapeutic potential. Animal experiments have shown that by pairing pharmacologically active drugs with exogenous stimuli, effective conditioned immune responses can be established.^182^ Subsequent exposure to an exogenous stimulus alone can mimic the effects of the drug, such as activating an immune response. One study found that after establishing a classical conditioning paradigm by pairing polyinosinic:polycytidylic acid (poly I:C) with a camphor odor, exposure to the camphor odor alone significantly extended the median survival of tumor-bearing mice and led to long-term tumor-free survival in a subset of animals, whereas the odor alone had no such effect.^182,183^ Chemotherapeutic agents themselves may suppress immune function.^184^ Through repeated pairing with such drugs, contextual cues in the chemotherapy clinic, acting as conditioned stimuli, can also lead to suppressed immune function in patients upon exposure to the environment alone.^185,186^ These findings highlight the importance of behavioral therapies and combined treatment strategies in cancer therapy. The combined use of behavioral interventions and immunosuppressive drugs (such as chemotherapeutic agents) can lead to behaviorally induced immunosuppression, which is termed conditioned immunosuppression.^187,188^ This raises new considerations for the design of associated clinical trials: treatment history must be thoroughly evaluated to avoid interference from non-pharmacological factors, such as conditioned immunosuppression.

### The role of the central nervous system in neuro-immune interactions of tumors

#### Neuro-immune interactions between the central nervous system and peripheral tumors

As an advanced integrative hub of neuro-immune regulation, the brain has increasingly been recognized for its central coordinating role in recent years. This section focuses on two key scientific questions. First, it investigates the biological mechanisms through which tumors and tumor-induced macroenvironmental remodeling function as interoceptive signals in brain-body communication and explores how the CNS perceives and integrates these pathological signals. Second, from a systemic disease perspective, it examines how the tumor macroenvironment impacts neuro-immune homeostasis in the CNS.

First, the CNS integrates and processes sensory inputs that elicit peripheral immune responses. As previously described, tumor cells in various cancer types secrete LIF and GAL3, which activate specific brain regions, including the PVN, suprachiasmatic nucleus, vestibular nuclei, and trigeminal motor nuclei. This neural activation promotes the production of MDSCs through sympathetic signaling, accelerating tumor progression.^16^ These findings demonstrate that tumors can communicate with the CNS via secreted proteins, functioning as critical interoceptive signals in brain-body communication. This process activates anti-inflammatory reflex pathways and establishes an immunosuppressive microenvironment favorable for tumor progression. In mouse models of pancreatic ductal adenocarcinoma and colorectal adenocarcinoma, the TME can specifically activate catecholaminergic neurons in the ventrolateral medulla (VLM). As a key regulatory hub of sympathetic neural signaling, the VLM, when activated, suppresses the antitumor immune function of CD8⁺ T cells via β-adrenergic receptor signaling, thereby promoting tumor immune evasion and disease progression.^189^ In mouse models of lung cancer, colorectal adenocarcinoma, fibrosarcoma, and melanoma, the TME has been shown to specifically activate hypothalamic neurons, which in turn promote the release of α-melanocyte-stimulating hormone (α-MSH) from the pituitary into the peripheral circulation through HPA regulation. This tumor-induced neuroendocrine response markedly enhances the expansion and activation of MDSCs in the bone marrow via its receptor MC5R.^190^ Furthermore, the insula encodes immunological memory of chronic inflammation, raising the question of whether similar mechanisms govern tumor-associated immune memory and subsequent immune phenotypes, an area warranting further investigation.^85^ The nervous system also modulates other characteristics of the macroenvironment, including metabolic states. During energy deficit, hypothalamic agouti-related peptide (AgRP) neurons suppress circulating Ly6C^hi^ monocyte levels.^191^ Dysregulation of the CNS, the central hub of the neuro-immune regulatory network, profoundly alters the dynamic balance of neuro-immune regulation through multiple pathways. Recent studies have uncovered specific mechanisms by which psychological stress modulates the TME via neuroendocrine pathways. Chronic psychological stress markedly activates the SNS, leading to excessive release of NE.^192^ This neurotransmitter binds to ADRB2 on tumor cells, significantly downregulating the expression of the RNA m6A demethylase ALKBH5. The resulting widespread disruption of the tumor cell epitranscriptome triggers a positive feedback loop of neural remodeling in the microenvironment that promotes tumor progression.^192^ Furthermore, depression, stress, and anxiety are common mood disorders among cancer patients and are associated with disease progression and the efficacy of immunotherapy.^193,194^ A previous review systematically summarized how tumors and the associated macroenvironment act as interoceptive signals that promote the development of depression and stress, as well as how these mood disorders, in turn, influence tumor progression.^195^ In brief, tumors promote the onset of depression and other mood disorders by hijacking the sympathetic-adrenal-medullary (SAM) axis, the HPA axis, and autonomic neural pathways, as well as by altering the composition of the gut microbiota. Depression, in turn, modulates the immune landscape of tumors through the neuroendocrine release of stress-associated immunomodulatory molecules. Beyond psychological states, recent evidence suggests that neurodegenerative conditions also influence tumor immunity. For instance, a study investigated the immune landscape in patients with Alzheimer’s disease (AD) and found that AD-associated amyloid precursor protein (APP) can activate the ceramide-fumarate metabolic axis, effectively protecting senescent T cells from excessive mitophagy and thereby preserving their antitumor immune function.^196^ This finding offers a novel perspective for understanding the complex relationship between neurodegenerative diseases and tumor immunity.

Tumors can influence the neuro-immune homeostasis of the CNS. As a systemic disease, cancer can reshape the phenotypes of multiple organs and disrupt neuro-immune axes.^197^ A growing body of evidence indicates that tumors can interfere with neuro-immune homeostasis in the CNS, leading to CNS dysfunction. First, tumors can induce inflammatory responses within the CNS, resulting in neurological symptoms such as circadian rhythm disruption. In a mouse model of liver cancer, tumors upregulated the activity of microglia and oxidative stress markers in the hypothalamic suprachiasmatic nucleus, thereby altering neuronal activity.^198^ This peripherally induced central neuroinflammation has been demonstrated to alter sleep patterns.^199,200^ Second, tumor-induced neuroinflammation in the CNS has been implicated in the development of mood disorders. Inflammatory cytokines such as IL-6 have been shown to play critical roles in tumor-associated depression and apathy.^201,202^ In addition, tumors can remodel the systemic metabolic landscape through neuro-immune mechanisms. For example, CCL2-driven inflammation in the dorsal motor nucleus of the vagus (DMV) can disrupt vagal function, suppress hepatic HNF4α expression, and trigger metabolic dysregulation and cachexia.^180^ Notably, tumor-mediated immunoregulation of the CNS may also contribute to the establishment of premetastatic niches. In melanoma and breast cancer models, tumor-secreted lipocalin-2 activated astrocytes and promoted the recruitment of inflammatory cells to the brain, thereby creating a favorable microenvironment for brain metastasis.^203^ In summary, current studies have focused mainly on tumor-induced neuroinflammation and its multifaceted consequences: (1) inducing neurological dysfunction such as insomnia and depression, (2) impairing the function of central neural regulatory hubs, and (3) shaping the premetastatic niche in the brain. These findings provide new insights into the systemic effects of cancer and offer a theoretical basis for developing therapeutic strategies targeting neurological complications.

#### Neuro-immune interactions in central nervous system tumors

Complex neuro-immune interactions within the CNS not only maintain its normal physiological functions but also shape a distinct immune microenvironment. In CNS malignancies, this microenvironment undergoes substantial remodeling, exerting critical effects on tumor initiation, progression, and therapeutic responses.^204,205^ Myeloid cells, represented primarily by microglia, constitute the major immune population in the brain and account for over 50% of infiltrating cells within the glioma immune microenvironment.^206,207^ Single-cell analyses have identified four major transcriptional programs in glioma-associated myeloid cells, among which the microglial inflammatory program is responsible for recruiting lymphocytes and is particularly enriched in IDH-mutant tumors.^207^ Notably, although this transcriptional program is specific to brain tumors, peripherally infiltrating myeloid cells can also be reprogrammed by the TME to acquire this phenotype. Neuronal activity can influence both tumor cells and immune cells through multiple molecular pathways. For example, neuronal activity regulates the secretion of BDNF and the shedding of the synaptic adhesion protein neuroligin-3 (NLGN3), thereby promoting the initiation and growth of neurofibromin 1 (Nf1)-driven optic pathway gliomas (Nf1-OPGs).^208^ These tumors concurrently exhibit low expression of immune-related gene signatures. Notably, research on glioma-neuron circuits has shown that glioblastoma (GBM) regions with enhanced neuron-tumor connectivity exhibit immunosuppressive features mediated by thrombospondin-1 (TSP-1). TSP-1 knockout disrupted synaptic formation, reduced glutamatergic neuron hyperexcitability, restored antitumor immunity, and prolonged survival in murine models.^209^ Although this study did not report the specific mechanisms of neuro-immune interactions, it provides a potential sensitization strategy for GBM, a tumor with poor immunotherapy outcomes. These findings also offer a research strategy for tumor invasion of peripheral nerves, suggesting a focus on the potential roles of electrical and chemical synapses in peripheral nerve-immune interactions. Importantly, neuro-immune interactions are not unidirectional. Gliomas originate from neuroectodermal stem cells or progenitor cells.^210^ This implies that tumor cells may share certain biological characteristics with normal neurons and glial cells. For example, gliomas can integrate themselves into neural circuits via electrical or chemical synapses and influence tumor phenotypes and patient prognosis.^211^ Consequently, they may share similar patterns of neuro-immune interactions. Following an immune attack, GBM stem cells undergo stable transcriptional and epigenetic reprogramming, which enhances the recruitment of TAMs and induces the formation of an immunosuppressive microenvironment.^212^ This underscores that GBM cells are not merely passive targets of immune cell attacks but also actively leverage their neural origin characteristics to communicate with immune cells in the brain.

In addition to their direct engagement in neuro-immune interactions within the CNS, CNS tumors may also reshape systemic immune profiles by perturbing adjacent neuroregulatory centers that govern immune responses, thereby modulating antitumor immunity. Patients with gliomas frequently exhibit systemic immunosuppression, manifested by reduced numbers and impaired functions of lymphocytes, particularly T cells, along with concomitant defects in cellular immunity.^213–216^ Recent studies have begun to uncover potential mechanisms underlying this phenomenon. One study reported that brain tumors can co-opt the HPA axis, triggering a surge in glucocorticoids (GCs) and upregulation of CCR8 in T cells, which sequesters them within the bone marrow and impairs antitumor immunity.^217^ Another study reported that both primary intracranial tumors and brain metastases induce the internalization of S1P1 in T cells, thereby restricting their egress from the bone marrow, reducing systemic T-cell counts, and preventing their infiltration into the TME.^218^ Notably, this mechanism has not been reported in other cancer types and may represent a physiological barrier that restricts T-cell infiltration into the CNS. However, its role in the context of tumors remains largely unexplored.

Taken together, CNS tumors establish a distinct immune microenvironment through both their intrinsic neuro-immune interactions and their capacity to influence neuroregulatory centers that orchestrate immune homeostasis. Deciphering how CNS tumors exploit specific neuro-immune pathways may uncover novel therapeutic avenues for intracranial malignancies. In parallel, large-scale comparative studies between intracranial and peripheral tumors are warranted to delineate the conserved and divergent features of neuro-immune interactions across cancer types.

## Neuro-immune interactions in immune-related disorders

Immune-related disorders are broadly classified into three categories on the basis of their pathogenesis and clinical manifestations, including allergic diseases, autoimmune diseases, and immunodeficiency diseases.^219^ Allergic diseases serve as a paradigmatic example of the three-tiered regulatory mechanism of neuro-immune interactions. Research on autoimmune diseases has focused more extensively on the dysregulation of local neuro-immune crosstalk. In contrast, the mechanisms underlying neuro-immune crosstalk in immunodeficiency diseases remain poorly understood.

### Local immune interactions in immune-related disorders

#### Allergic diseases

**Fig. 3:**
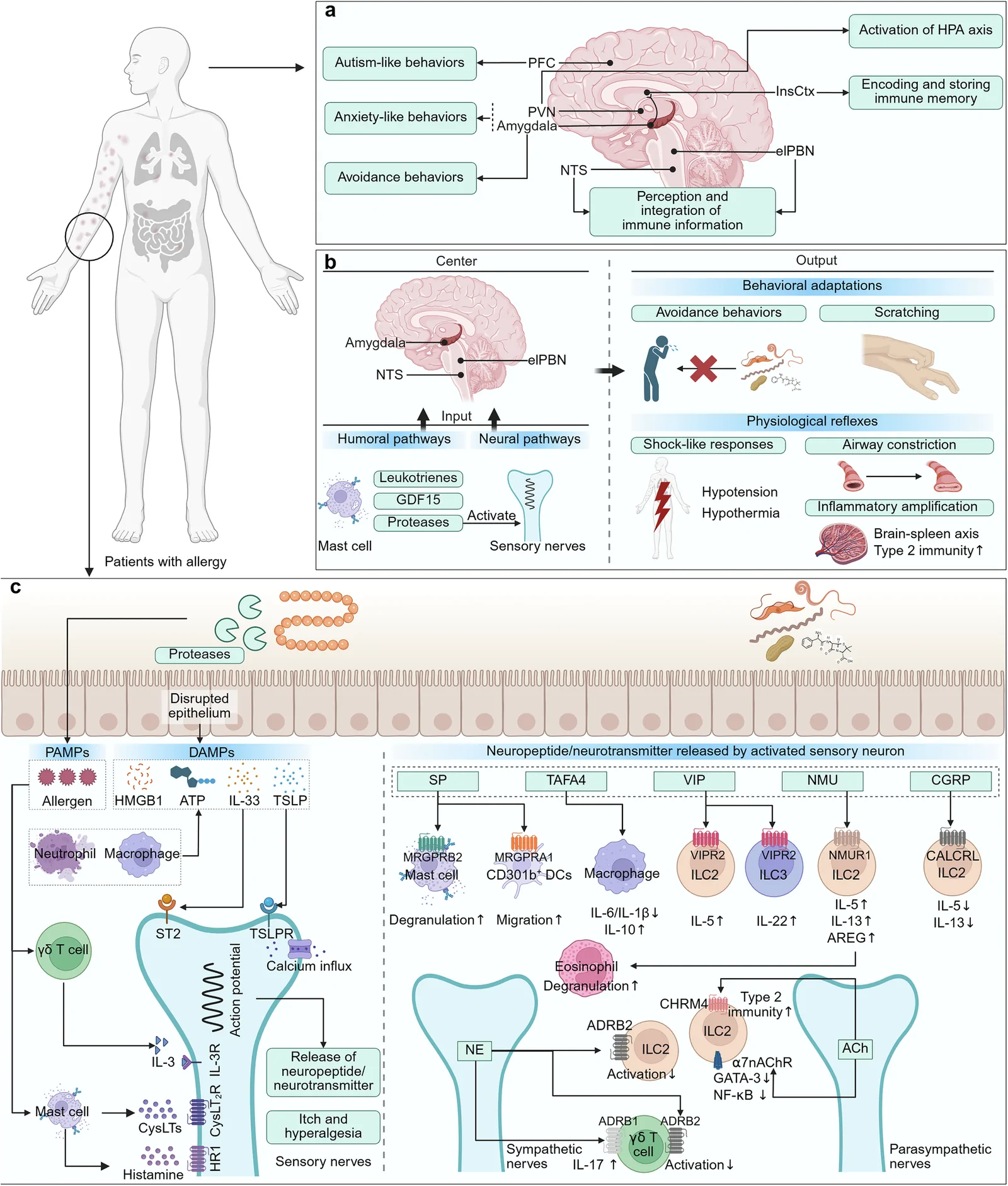
Neuro-immune interactions in allergic diseases. Neuro-immune interactions in allergic diseases are closely associated with mucosal tissues. **a** The brain integrates immune signals via regions including the NTS and InsCtx, which encode immune memory. Specific regions mediate distinct behaviors: the PFC for autism-like behaviors, the PVN and amygdala for anxiety-like behaviors, and the elPBN for avoidance behaviors. The hypothalamic-pituitary-adrenal axis modulates systemic responses. **b** Allergic responses are regulated by neuro-immune reflexes. Inputs include humoral signals and sensory nerves. Central hubs include the NTS and amygdala. Outputs induce behavioral adaptations and physiological reflexes. **c** Sensory nerves mediate allergic symptoms. Immune cells in the mucosa release cytokines that activate sensory neurons and trigger itching and allodynia. Sensory neurons, sympathetic nerves, and parasympathetic nerves release neurotransmitters to regulate immune function. The figure was created with BioRender.com. ACh acetylcholine, ADRB1 β1-adrenergic receptor, ADRB2 β2-adrenergic receptor, AREG amphiregulin, CGRP calcitonin gene-related peptide, CHRM4 cholinergic receptor muscarinic 4, DAMPs damage-associated molecular patterns, elPBN external lateral parabrachial nucleus, GATA-3 GATA binding protein 3, GDF15 growth differentiation factor 15, Glu glutamate, HR1 histamine receptor-1, ILC2 innate lymphoid cell 2, ILC3 innate lymphoid cell 3, InsCtx insular cortex, NE norepinephrine, NMU neuromedin U, NTS nucleus tractus solitarius, PAMPs pathogen-associated molecular patterns, PFC prefrontal cortex, PVN paraventricular nucleus, SP substance P, TAFA4 TAFA chemokine-like family member 4, TSLP thymic stromal lymphopoietin, TSLPR thymic stromal lymphopoietin receptor, CysLTs cysteinyl leukotrienes, NF-κB nuclear factor kappa B

From a local perspective, allergic diseases are mediated by complex neuro-immune mechanisms that operate across multiple mucosal and epithelial barriers. Human mucosal barrier tissues and the skin are densely innervated (Fig. 3).^220^ These regions continuously monitor foreign antigens through neuro-immune crosstalk to sustain homeostasis.^221,222^ Dysregulation of this system contributes to mucosal allergic and inflammatory disorders such as asthma, allergic rhinitis, and inflammatory bowel disease. Local neuro-immune interactions encompass the perception of allergens and the regulation of localized allergic reactions.

Sensory neurons within barrier tissues, such as the skin, respiratory tract, and intestine, can sense both allergens and signals of tissue damage elicited by them. Allergens such as house dust mites, cockroaches, and *Alternaria* species possess protease activity that damages epithelial cells and induces the release of damage-associated molecular patterns (DAMPs), including HMGB1 and ATP.^223,224^ These DAMPs activate DRG neurons via receptors such as P2X3 and receptor for advanced glycation end-products (RAGE), leading to neuronal depolarization and neuropeptide release.^224,225^ Epithelial-derived alarmins, including IL-33 and thymic stromal lymphopoietin (TSLP), can also act directly on neurons.^226,227^ Specifically, TSLP induces acute itch and neuronal depolarization via its receptor TSLPR, while IL-33 signals through the ST2/IL-1RAcP complex expressed in DRG neurons to promote calcium influx via TRPV1/TRPA1 channels, thereby contributing to chronic itch^228–230^ From an indirect sensory perspective, activated immune cells such as macrophages and neutrophils can release ATP, which further activates neurons.^231,232^ Recent studies have demonstrated that upon allergen exposure, skin-resident γδ T cells secrete IL-3 to activate JAK2-STAT5 signaling in sensory neurons, inducing pruritus and localized inflammation.^8^

Sensory and autonomic nerves precisely regulate immune responses through the release of various neuropeptides and neurotransmitters. Upon stimulation by allergens or tissue injury, sensory neurons rapidly release SP or TAFA4.^233–237^ SP can induce mast cell degranulation via MRGPRB2 and promote the migration of CD301b⁺ dendritic cells through MRGPRA1, whereas TAFA4 suppresses the secretion of IL-6 and IL-1β by macrophages while enhancing IL-10 production to drive local anti-inflammatory responses during ultraviolet-induced skin injury. VIP⁺ sensory neurons promote ILC2-derived IL-5 secretion and ILC3-derived IL-22 secretion via VIPR2.^238,239^ In contrast, CGRP⁺ sensory neurons inhibit ILC2 production of IL-5 and IL-13 through the RAMP1/CALCRL pathway.^240^ Studies have demonstrated that during helminth infection, a subset of pulmonary ILC2s co-expressing IL-5 and CALCA is present.^241^ CGRP, in synergy with IL-33 and NMU, promotes IL-5 expression while suppressing IL-13 production and ILC2 proliferation.^241^ Consistent with observations in tumors, these results underscore the immunosuppressive role of CGRP. Sympathetic nerve terminals release NE, which can either activate γδ T cells through the CaMK2γ/ADRB1/NF-κB/p38/IL-17 axis to exacerbate psoriatic inflammation or suppress the inflammatory effects of ILC2s and γδ T cells via ADRB2.^57,242,243^ ChAT⁺ cholinergic neurons exert bidirectional regulatory effects. Their release of NMU potently activates NMUR1⁺ ILC2s to induce the secretion of IL-5, IL-13, and amphiregulin (AREG) and to stimulate eosinophil degranulation.^244,245^ Recent work has shown that postprandial parasympathetic activation in the lungs enhances type 2 immunity by acting on CHRM4^+^ ILC2s, potentially exacerbating asthma.^9^ Intriguingly, ILC2s express α7nAChR, and pharmacological activation of this receptor attenuates ILC2 effector function by suppressing the key type 2 transcription factor GATA-3 and inhibiting NF-κB-mediated inflammatory signaling.^246^ Collectively, these pathways form a neurocentric bidirectional regulatory network that balances “proinflammatory drive” and “anti-inflammatory brake” mechanisms to finely tune the intensity and duration of allergic and inflammatory responses. Once an allergic response is initiated, immune cells such as mast cells and ILC2s rapidly convert incoming neural signals into self-activating cues and, in turn, release inflammatory mediators that establish a self-amplifying neuro-immune positive feedback loop. Degranulated mast cells release histamine and cysteinyl leukotrienes, which directly bind to H_1_R and CysLT_2_R on sensory neurons, lowering their action potential threshold and promoting further release of SP and CGRP.^247,248^ Moreover, the IgE-FcεRI complex is expressed on the membranes of vagal jugular-pulmonary sensory neurons. Upon re-exposure to allergens, IgE crosslinking triggers rapid neuronal SP release to amplify immune responses, effectively bypassing the conventional sensitization phase and compressing the “secondary response” into a fast neuron-mediated reflex.^249^ Through this iterative process, close neuro-immune cooperation amplifies local allergic reactions.

#### Autoimmune diseases

##### Autoimmune disorders of the nervous system

**Fig. 4:**
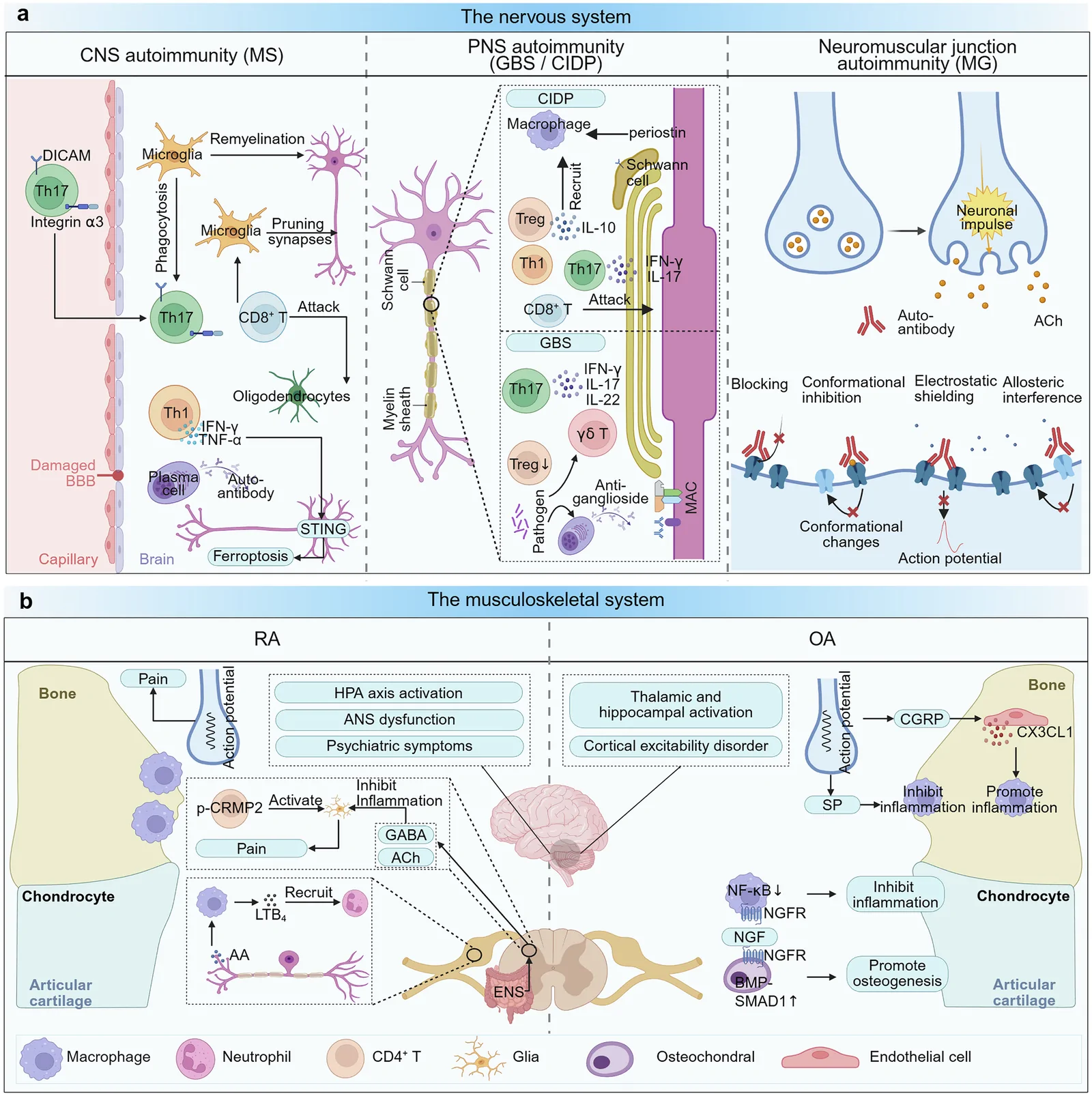
Neuro-immune interactions in autoimmune diseases. Neuro-immune interactions participate in representative autoimmune diseases. **a** Autoimmune disorders of the nervous system involve processes such as BBB disruption, immune cell activation, and neuronal injury. **b** Neuro-immune crosstalk in RA and OA includes shared features such as local macrophage regulation, pain signaling, and central nervous system involvement. The figure was created with Biorender.com. ACh acetylcholine, AA arachidonic acid, BBB blood-brain barrier, BMP bone morphogenetic protein, CGRP calcitonin gene-related peptide, CIDP chronic inflammatory demyelinating polyneuropathy, CNS central nervous system, CX3CL1 C-X3-C motif chemokine ligand 1, ENS enteric nervous system, GABA gamma-aminobutyric acid, GBS Guillain-Barré syndrome, HPA axis hypothalamic-pituitary-adrenal axis, LTB_4_ leukotriene B_4_, MAC membrane attack complex, MG myasthenia gravis, MS multiple sclerosis, NF-κB nuclear factor kappa B, NGF nerve growth factor, NGFR nerve growth factor receptor, OA osteoarthritis, p-CRMP2 phosphorylated collapsin response mediator protein 2, PNS peripheral nervous system, RA rheumatoid arthritis, SP substance P, STING stimulator of interferon genes, Th17, T helper 17 cells. TNF-α tumor necrosis factor alpha

Autoimmune disorders of the nervous system provide a direct illustration of neuro-immune interactions. Broadly, these effects can be summarized in two dimensions: first, the impact of infiltrating immune cells on the nervous system during disease onset and progression, and second, the alteration of pre-existing neuro-immune homeostasis and its implications in disease (Fig. 4).

**Multiple sclerosis**

Multiple sclerosis (MS) is one of the most common chronic inflammatory, demyelinating, and neurodegenerative diseases of the CNS.^250^ Among the currently proposed pathogenic mechanisms, T cells and B cells are considered key drivers of MS, playing a critical role, particularly in the formation of focal lesions.^251^ In addition, tissue-resident myeloid cells, such as microglia, which serve as primary regulators of neuro-immune homeostasis in the CNS, are also involved in various pathophysiological processes, including remyelination.^252^ Therefore, MS is characterized not only by pathological damage mediated by the adaptive immune system against neural tissue but also by the dysregulation of intrinsic neuro-immune interactions during disease onset and progression.

In the pathogenesis of MS, T cells are widely recognized as central players. Across different disease stages and lesion phases, including acute MS cases of extremely short duration, CD8⁺ T cells predominate.^253^ Within active lesions, a subset of infiltrating CD8⁺ T cells exhibits proliferative activity and cytotoxic effector functions.^254^ In experimental autoimmune encephalomyelitis (EAE), CD4⁺ T-cell infiltration appears sparse, yet the transfer of myelin-reactive CD4⁺ T cells suffices to induce disease, underscoring their initiating role.^255,256^ Th1 cells are thought to augment the cytotoxic capacity of CD8⁺ T cells.^257^ In the EAE model, lesion sites exhibit elevated expression of Th1-associated cytokines, including IFN-γ, TNF-β, TNF-α, and IL-12, which may exacerbate local inflammation.^258^ Clinical studies further revealed that the circulating Th1/Th2 cell ratio is positively correlated with disease severity.^259^ Although IFN-γ has long been regarded as a key pathogenic factor in EAE, recent findings suggest that it may also contribute to disease resolution by inducing the conversion of Treg cells into Th1-like Treg subsets with reparative functions.^260,261^ Meanwhile, Th17 cells have been strongly implicated in tissue inflammation and are increasingly recognized as dominant drivers in EAE pathogenesis.^262,263^ For example, deletion of the enhancer RORCE2 within the RORγt gene impairs Th17 cell differentiation and attenuates EAE severity.^264^ Dual immunoglobulin domain-containing cell adhesion molecule (DICAM), expressed by Th17 cells, facilitates blood-brain barrier (BBB) penetration during neuroinflammation, and blockade with a monoclonal antibody against DICAM inhibits this process and ameliorates disease.^265^ Another study demonstrated that Th17 migration into the CNS depends on integrin α3, the absence of which causes CD4⁺ T cells to accumulate in the perivascular spaces of the BBB.^266^

In addition to T cells, B-cell dysregulation is critically involved in MS pathogenesis and progression, as evidenced by (1) the production of autoantibodies, with detectable abnormal immunoglobulins in the cerebrospinal fluid, (2) the secretion of proinflammatory cytokines, (3) the release of soluble toxic mediators that damage oligodendrocytes and neurons, (4) antigen presentation to T cells, (5) contribution to the formation of tertiary lymphoid structures within the CNS, and (6) harboring Epstein-Barr virus.^267,268^

Tissue-resident myeloid cells, such as microglia, play essential roles in sustaining immune homeostasis in both the CNS and PNS, serving as key participants in neuro-immune interactions under physiological conditions.^65,269^ During normal development, microglia regulate synaptic density and circuit connectivity by clearing senescent cells.^270^ In EAE, neuroinflammation triggers myeloid cells to phagocytose infiltrating Th17 lymphocytes through a process mediated by activation-dependent lectins and their T-cell surface ligand N-acetyl-D-glucosamine (GlcNAc), suggesting a potential defense mechanism for maintaining CNS immune privilege.^271^ Microglia can also polarize toward reparative states and promote remyelination in MS lesions.^252^ Conversely, in demyelinating disease models, microglia may aberrantly enhance synaptic pruning, leading to neurological dysfunction, a process reported to be triggered by CD8⁺ T cells.^272,273^ In glioma-associated neuro-immune interactions, microglia with proinflammatory transcriptional programs recruit T-cell infiltration.^207^ Similarly, microglia exhibiting inflammatory transcriptional signatures have been identified in MS, although their direct role in T-cell recruitment remains unclear.^274^ Overall, microglia may delay disease progression or promote repair by sustaining neuro-immune homeostasis under physiological conditions. However, under pathological circumstances, they can be hijacked into proinflammatory phenotypes, engaging closely with T cells and other immune populations to drive disease progression. In addition to tissue-resident myeloid cells, a study demonstrated that autoreactive T cells in MS selectively home to the bone marrow, where they promote myelopoiesis via the CCL5-CCR5 signaling axis.^275^ This, in turn, exacerbates CNS neuroinflammation in EAE, establishing a broader regulatory circuit that links neural antigens, circulating T cells, and bone marrow responses.

Interestingly, neurons themselves can contribute to inflammatory processes by upregulating immune-related gene expression. Under conditions of glutamate toxicity, neuronal STIM1 dissociates from STING, leading to noncanonical activation of the STING pathway.^276^ This, in turn, induces the autophagic degradation of GPX4, ultimately driving ferroptosis and exacerbating neuroinflammation. This mechanism highlights that neurons are not merely passive targets but also capable of actively modulating the local immune microenvironment.

**Guillain-Barré syndrome/chronic inflammatory demyelinating polyneuropathy**

Guillain-Barré syndrome (GBS) and chronic inflammatory demyelinating polyneuropathy (CIDP) are inflammatory demyelinating diseases affecting the PNS that variably impair motor, sensory, and autonomic fibers.^277,278^ Unlike MS, GBS and CIDP are autoimmune disorders of the PNS that exhibit immunological features distinct from those of the CNS. An intact peripheral nerve comprises neurons, Schwann cells, fibroblasts, pericytes, endothelial cells, and resident macrophages in close proximity to mast cells and other tissue-resident immune populations, suggesting that neuro-immune interactions in GBS and CIDP may differ from those in MS.^279^

Both humoral immunity, primarily mediated by B cells, and cellular immunity, mediated by T cells, contribute to GBS pathogenesis.^280^ Most GBS cases are associated with preceding infections, particularly *Campylobacter jejuni*, of which lipooligosaccharide (LOS) can cross-react with host gangliosides through molecular mimicry, eliciting autoreactive antibodies.^281,282^ γδ T cells, capable of recognizing non-protein antigenic epitopes, also participate in ganglioside recognition and disease progression.^283^ Single-cell transcriptomics further revealed that some GBS patients lacking anti-ganglioside antibodies nonetheless harbor autoreactive T cells targeting PNS-specific myelin antigens such as P0, P2, and PMP22, suggesting that the antigen specificity of different T-cell clones may underlie disease heterogeneity.^284^ Studies have found a reduction in peripheral Tregs in acute GBS, implicating their potential role in disease initiation, although the underlying mechanisms remain to be fully elucidated.^285–287^ CD4⁺ T cells can differentiate into Th1/Th17 subsets, producing proinflammatory cytokines, including IFN-γ, IL-17, and IL-22, thereby exacerbating neuroinflammation.^288,289^ Recent evidence indicates that P2X7 receptor inhibition alleviates experimental autoimmune neuritis by modulating CD4⁺ T-cell differentiation and suppressing NLRP3 inflammasome activation.^290^ Complement activation also plays a key role in GBS, leading to the formation of the membrane attack complex (MAC) and direct damage to myelin or axons, a mechanism less prominent in MS.^291^

CIDP differs from GBS primarily in its chronic, relapsing course, indicating sustained involvement of inflammatory immune mechanisms.^292^ Similar to those in GBS, CD4⁺ T cells are central to CIDP, with increased Th1 cells producing IFN-γ and Th17 cells producing IL-17 detected in patient peripheral blood and cerebrospinal fluid.^293,294^ Mice deficient in IFN-γ or IL-17 are resistant to PNS autoimmunity.^295^ Notably, CIDP patient nerves exhibit upregulated IL-10 expression from Tregs, which promotes CD4⁺ T-cell migration from lymph nodes to peripheral nerves via an S1PR1-dependent mechanism, recruits macrophages, and induces Schwann cells apoptosis through the FAS pathway.^296,297^ Unlike in GBS, CD8⁺ T cells play a prominent role in CIDP, directly mediating axonal or myelin injury.^298,299^ Macrophages represent the main infiltrating immune cells in CIDP nerve biopsies, driving demyelination through phagocytosis, with Schwann cells promoting macrophage recruitment via the upregulation of periostin, and macrophage depletion delays disease progression.^300,301^ Analogous to CNS microglia, macrophages exhibit dual functions, clearing debris while facilitating remyelination.^302,303^

**Myasthenia gravis**

Myasthenia gravis (MG), as a prototypical autoimmune disorder, exhibits distinctive pathological features.^304^ Its core mechanism involves antibody-mediated immune attacks targeting postsynaptic AChRs, muscle-specific kinase, and other AChR-associated proteins. By directly binding to neurotransmitter receptors and related functional proteins, these autoantibodies impair neuronal states.^304^ It provides an additional perspective for understanding neuro-immune interactions.

A recent study resolved high-resolution structures of six monoclonal antibody Fab fragments derived from different MG patients in complex with the AChR and combined these findings with electrophysiological assays.^305^ This work revealed four molecular mechanisms by which autoantibodies directly disrupt AChR ion channel function: (1) blocking mechanism: antibodies physically occupy the ACh binding sites, preventing neurotransmitter engagement; (2) conformational inhibition mechanism: antibodies do not occlude the binding sites but suppress local ACh-induced conformational changes, thereby inhibiting channel activation; (3) electrostatic shielding mechanism: antibodies bind around the channel pore, altering the surface electrostatic distribution, neutralizing cation-attracting charges, and diminishing transmembrane ion flux; and (4) allosteric interference mechanism: antibodies bind at subunit interfaces, disrupting essential conformational rearrangements required for the transition from the resting to the open state. These findings provide a direct atomistic-level explanation of how autoantibodies directly induce AChR dysfunction, thereby deepening our understanding of MG pathogenesis and offering a structural basis for the development of novel targeted therapeutic strategies. More broadly, this study highlights the fine-tuned regulation of neural function by immune molecules.

##### Autoimmune disorders of the musculoskeletal system

The musculoskeletal system comprises bones, skeletal muscles, and joints.^306^ This section focuses on two major disorders: rheumatoid arthritis (RA) and osteoarthritis (OA) (Fig. 4).

**Rheumatoid arthritis**

Pain is one of the most prominent clinical manifestations of RA.^307^ As a systemic chronic inflammatory disease, RA-induced pain involves both persistent activation of nociceptors and central sensitization mechanisms.^308,309^ At the local level, sensory neurons serve as key mediators of pain transmission. For example, studies using the K/BxN serum transfer arthritis model have shown that capsaicin-sensitive sensory neurons not only contribute to mechanical allodynia but also exert anti-inflammatory effects through the release of somatostatin.^310^

Notably, RA patients often report pain symptoms that do not fully correlate with disease activity. Recent findings suggest that in the context of inflammatory pain, arachidonic acid (AA) released from DRG neurons can be metabolized by macrophage-expressed 5-lipoxygenase into leukotriene B4 (LTB_4_), thereby recruiting neutrophils.^311^ Efficient clearance of apoptotic neutrophils requires timely macrophage-mediated efferocytosis. This study further demonstrated that activating the MerTK receptor enhances macrophage efferocytic capacity and alleviates pain. Another study in a collagen-induced arthritis (CIA) model revealed that phosphorylation of collapsin response mediator protein 2 (CRMP2) augments CD4⁺ T-cell migration and trans-endothelial invasion, promoting their infiltration into the spinal dorsal horn, where they activate glial cells and trigger pain behaviors.^312^

**Osteoarthritis**

OA is a prevalent degenerative joint disorder characterized by progressive cartilage degradation and chronic pain.^313^ While OA is traditionally considered a degenerative disease rather than an autoimmune disease, alterations in synovial lymphoid structures, circulating autoantibodies, and memory T-cell and B-cell populations have been recorded in OA.^314^ Therefore, we will discuss OA in this section. Emerging evidence highlights the involvement of neural pathways in both pain signal transmission and local immune response modulation. Recent studies further suggest potential interactions between OA pathology and the brain.^315–317^

Clinically, OA patients not only experience localized pain but also exhibit heightened sensitivity to thermal, mechanical, and pressure stimuli.^318,319^ NGF levels are positively correlated with OA pain severity, yet NGF inhibition may exacerbate bone destruction. Studies indicate that NGF suppresses the NF-κB pathway in immune cells via the NGFR pathway, thereby attenuating inflammation. Concurrently, NGFR modulates the bone morphogenetic protein (BMP)-suppressor of mothers against the decapentaplegic (SMAD1) signaling axis to promote bone formation while inhibiting receptor activator of nuclear factor-κ B ligand (RANKL)-dependent osteoclastogenesis, fostering a type II immune response conducive to tissue repair.^320^ While prior research has predominantly examined the influence of immune cells or systemic inflammation on neuronal signaling, particularly in the context of OA-related pain, the dense innervation of bone and joint regions also shapes local immune responses. Sensory neurons release neuropeptides that modulate immune activity. For instance, SP induces an anti-inflammatory macrophage phenotype in the RA mouse model. Similarly, intra-articular SP-releasing hydrogels reduce joint inflammation in an OA mouse model.^321,322^ Conversely, CGRP promotes CX3CL1 secretion from endothelial cells, promoting the inflammatory phenotype of macrophages. Notably, CGRP inhibitors have demonstrated efficacy in alleviating joint inflammation.^323,324^

##### Autoimmune disorders of the endocrine system

T1DM represents a prototypic autoimmune disease that differs fundamentally from mucosal allergic disorders. T1DM primarily targets the pancreas, a solid organ densely innervated by autonomic nerves, and lacks the antigen-rich microenvironment characteristic of mucosal surfaces.^325^ The current pathogenic model attributes T1DM to immune-mediated destruction of pancreatic β-cells.^326^ However, emerging evidence has demonstrated significant sympathetic neurodegeneration and immune cell infiltration within pancreatic nerves in T1DM patients, suggesting a critical role for local neuro-immune crosstalk in T1DM.^327^ Sympathetic neurodegeneration may be partially explained by immune targeting of key T1DM autoantigens, including glutamic acid decarboxylase 65 (GAD65) and protein tyrosine phosphatases (PTPs), which are also expressed in neurons.^328–330^ Pharmacological blockade of β-adrenergic receptors suppresses IL-1β while increasing IL-10 in pancreatic tissue.^331^ Targeted electrical stimulation of sympathetic nerves innervating pancreatic lymph nodes reduces the T1DM incidence in non-obese diabetic mice, attenuating LPS-induced proinflammatory cytokine production and autoreactive T-cell proliferation.^332^ These findings suggest that sympathetic nerves exert divergent effects within the pancreatic parenchyma and secondary lymphoid organs. A recent study demonstrated that sympathetic neurons, via ADRB2-dependent signaling, promote the gut homing of ILC2s. These ILC2s subsequently migrate to the pancreas, where they secrete cytokines that stimulate pancreatic α-cells to produce glucagon, thereby enhancing hepatic gluconeogenesis and sustaining systemic glucose homeostasis.^333^ Together, these insights not only advance our understanding of T1DM pathogenesis but also identify promising therapeutic targets. Targeting neuro-immune pathways may yield innovative treatments for T1DM and related metabolic disorders.

Overall, the aforementioned diseases present distinct patterns of local neuro-immune interactions. OA and RA are autoimmune diseases characterized primarily by chronic pain, highlighting the role of nociceptors in such autoimmune conditions. It is worth noting that pain is generally accompanied by the activation of the SNS.^334^ The role of the SNS in these typical painful autoimmune diseases needs further investigation. In the case of T1DM, attention has been focused on how neural regulation of tissue-resident immune cells affects normal pancreatic function and the local inflammatory status. Additionally, other autoimmune diseases with neuro-immune interaction mechanisms are worth discussing. SLE is an autoimmune disease in which approximately half of patients exhibit neuropsychiatric symptoms and are often diagnosed with neuropsychiatric SLE.^335^ In the classic SLE model using MRL-lpr mice, chronic stress was found to activate microglia in the medial prefrontal cortex, which in turn activated neurons in this region through IL-12 and IL-23, inducing symptoms of neuropsychiatric SLE.^336^ It is worth mentioning that the MRL-lpr mouse model is also used to study RA, suggesting that similar patterns of psychiatric abnormalities may exist in these autoimmune diseases.

#### Immunodeficiency diseases

Defects in one or more components of the immune system can lead to immunodeficiency disorders, which are broadly categorized into primary immunodeficiency diseases (PIDs) and secondary immunodeficiency diseases (SIDs).

**Fig. 5:**
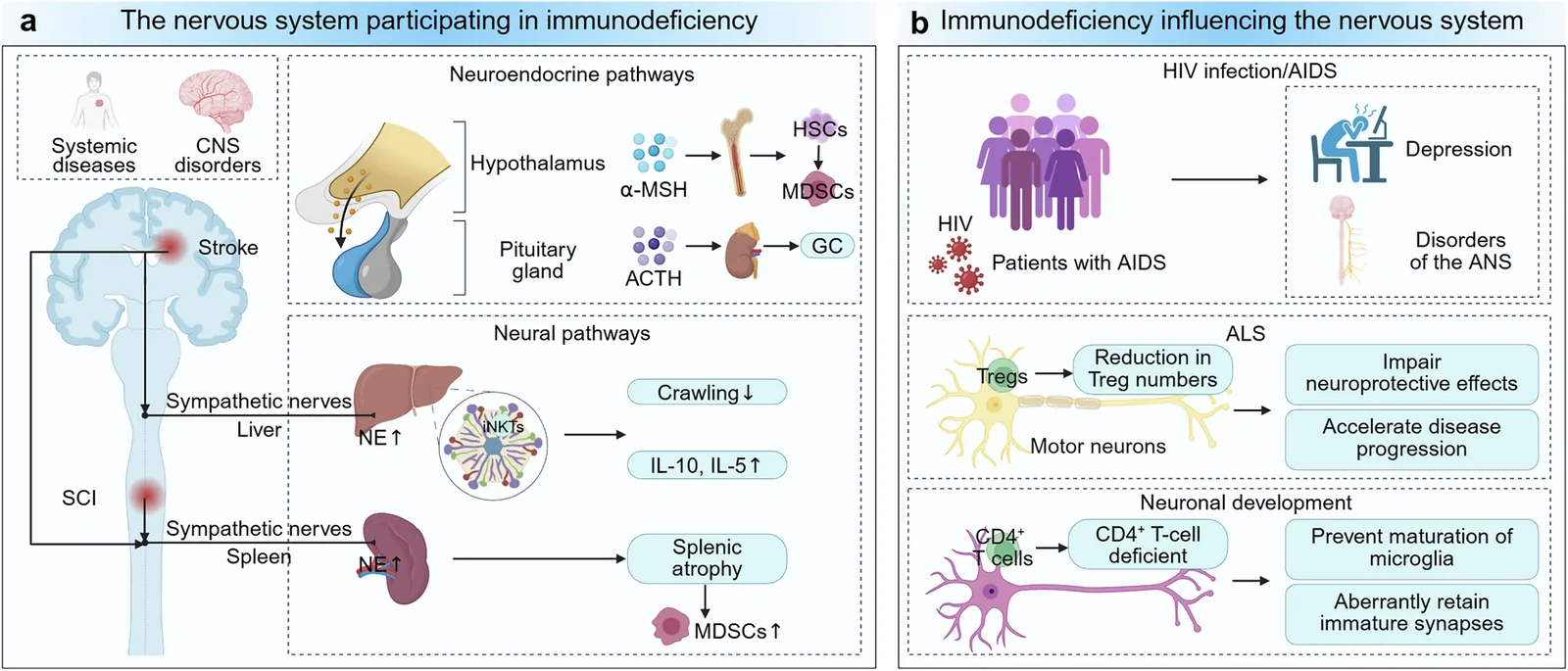
Neuro-immune interactions in immunodeficiency diseases. The nervous system is involved in the development of secondary immunodeficiency. **a** In systemic diseases such as cancer, CNS injury induces systemic immunosuppression by hijacking neurohumoral axes such as the HPA axis and activating sympathetic outflow to the liver and spleen. **b** Conversely, immune dysfunction impairs CNS development and contributes to ALS progression and AIDS-associated neuropsychiatric manifestations. The figure was created with BioRender.com. ACTH adrenocorticotropic hormone, AIDS acquired immunodeficiency syndrome, ALS amyotrophic lateral sclerosis, ANS autonomic nervous system, α-MSH alpha-melanocyte-stimulating hormone, CNS central nervous system, HPA hypothalamic-pituitary-adrenal, HSC hematopoietic stem cell, iNKT invariant natural killer T-cell, MDSCs myeloid-derived suppressor cells, NE norepinephrine, SCI spinal cord injury

Neural innervation of central and peripheral immune organs raises the intriguing possibility that the nervous system may contribute to the pathogenesis of PIDs by influencing immune organ development, although this hypothesis awaits experimental validation (Fig. 5). Meanwhile, phenotypic remodeling of immune organ innervation has been implicated in the development of SIDs under certain pathological conditions. In tumor models, for example, activation of the brain-spleen axis augments splenic sympathetic signaling, thereby expanding MDSCs and promoting systemic immunosuppression. In melanoma, tumors exploit the hypothalamic-pituitary axis to stimulate α-MSH secretion, which drives myelopoiesis in the bone marrow and culminates in profound immunosuppression.^190^ Moreover, peripheral neuropathy frequently arises as a complication of therapeutic interventions for diabetes and cancer. Notably, peripheral nerves innervate immune structures, including DRG projections to peripheral lymph nodes, suggesting that neural pathways may serve as a bridge between peripheral nerve damage and immune dysfunction in diabetes and cancer.^337^ These findings open new avenues for investigating how disease-associated neural alterations may mediate immunosuppression.^338,339^

### Neuro-immune reflexes in immune-related disorders

#### Allergic diseases

Neuro-immune reflexes tightly couple allergic immune activation to behavioral and physiological responses (Fig. 3). Allergic reactions frequently evoke avoidance behaviors. For example, antigen-specific immunity drives mice to actively avoid drinking water containing the immunogen.^340^ In allergic pneumonitis, persistent and appropriate antigen avoidance often facilitates disease improvement or stabilization.^341^ Recent work reported that ovalbumin (OVA)-sensitized mice exhibit pronounced avoidance of OVA and that oral OVA activates autonomic regulatory brain regions, including the nucleus tractus solitarius (NTS), the external lateral parabrachial nucleus (elPBN), and the CeA.^132,340^ Follow-up experiments revealed that intestinal mast cells are key relays of allergic signals and are capable of transmitting allergen-derived cues even before intestinal allergic inflammation emerges. Subdiaphragmatic vagotomy excludes vagal involvement, indicating that the signal is instead transmitted via a humoral route mediated by growth differentiation factor 15 (GDF15) and leukotrienes, thereby offering new perspectives for neuroimmunology. A contemporaneous study likewise underscored the role of mast cells in avoidance signaling and further delineated local intestinal mechanisms. Barrier disruption facilitates antigen entry and triggers type 2 immunity.^342^ Adaptive responses produce antigen-specific IgE that binds mast cells, enabling rapid avoidance upon re-exposure. Pharmacological blockade of FLAP-dependent leukotriene synthesis impaired this behavior, further substantiating leukotrienes as pivotal mediators. Allergy-induced avoidance behaviors reduce antigen exposure and may thus protect against allergic exacerbations. Another behavior closely linked to allergies, scratching, may also serve distinct physiological functions. Scratching activates TRPV1⁺ sensory neurons, induces SP release, and triggers mast cell degranulation, TNF secretion, and neutrophil recruitment, thereby amplifying inflammation.^343^ However, in a *Staphylococcus aureus* skin infection model, scratching-induced inflammation enhanced local host defenses and effectively lowered the bacterial burden. Thus, scratching aggravates inflammation in allergic contexts but may confer protection during infection by increasing local immunity.^343^

In addition to behavior, allergic reactions can trigger neuro-immune reflexes that produce shock-like physiological responses. Upon allergen activation, sensitized mast cells release not only vasoactive granular mediators but also chymase, which engages PAR1 on TRPV1⁺ sensory neurons.^36^ Activation of these neurons propagates downstream neural circuits, causing rapid declines in body temperature and blood pressure. One study reported that repeated inhalation of allergens in mice activates Dbh⁺ neurons in the NTS through mast cell, IL-4-dependent, and vagus nerve-dependent mechanisms.^344^ Viral tracing revealed that these Dbh⁺ NTS neurons project to the nucleus ambiguus and that neurons in the nucleus ambiguus are both necessary and sufficient to relay allergen-evoked signals downstream to postganglionic neurons that directly drive airway constriction. Another study found that allergen stimulation and exposure to fine particulate matter upregulate TRPV1 channels in trigeminal neurons and elevate brainstem metabolites such as glutamate and glutamine, ultimately exacerbating type 2 inflammatory responses in the spleen via the brain-spleen axis and thereby aggravating the pathological manifestations of allergic asthma.^345^

Collectively, neuro-immune reflexes represent a critical interface linking immune activation to behavioral adaptation and systemic physiological or pathological outcomes.

#### Autoimmune diseases

The anti-inflammatory reflex plays a valuable role in various autoimmune diseases (Fig. 4). In demyelinating autoimmune diseases, VNS has been shown to exert neuro-immune regulatory effects in a rat EAE model, significantly reducing disease severity, shortening disease duration, and suppressing key immunopathological processes such as T-cell infiltration and proinflammatory microglial activation.^10^ This indicates that the ANS participates in EAE pathogenesis and suggests that neuro-immune reflexes may play important roles in MS, offering new potential avenues for therapeutic intervention.

In RA, a recent investigation established a link between spinal immune homeostasis and local inflammation: in the CIA model, gut microbiota-derived histamine activated the enteric nervous system (ENS), which in turn increased the release of neurotransmitters such as ACh and GABA in the spinal cord.^346^ This process suppressed the inflammatory phenotype of microglia and reduced vascular leakage via the “gut-CNS-joint axis”, thereby attenuating both arthritis and pain. Collectively, these studies elucidate the complex mechanisms of RA pain at multiple levels, including peripheral nerves, immune cell infiltration, and central sensitization. Interestingly, a cohort study reported that truncal vagotomy was significantly associated with an increased risk of RA, but not OA, underscoring the specific role of autonomic neuro-immune reflexes in RA pathogenesis.^347^ Given the involvement of the vagus nerve in systemic anti-inflammatory reflexes, this finding further supports the characterization of RA as a systemic inflammatory disease.

### The role of the central nervous system in immune-related disorders

#### Allergic diseases

In the previous section, we systematically examined the role of neuro-immune reflexes in allergic diseases. We noted that allergens can function as sensory inputs within these reflex pathways, whereas the effectors may manifest as physiological or pathological changes in organs or systems regulated by the ANS or as antigen-directed avoidance behaviors (Fig. 3). This suggests that the brain regions participating in these reflexes likely include the brainstem and higher-order centers, highlighting the capacity of the brain to receive, integrate, and respond to allergy-related immune information. The brainstem, which houses the nuclei governing multiple autonomic reflexes, can mediate the influence of allergic responses on organ and systemic function by modulating autonomic outflow. As summarized above, local allergic responses can activate specific brainstem nuclei, such as the NTS and the elPBN, via humoral mediators such as GDF15 and leukotrienes or neural routes such as the vagus nerve.^340,342^

Allergic responses can engage higher-order brain regions. For example, OVA feeding in sensitized mice not only activates the NTS and elPBN but also strongly recruits the CeA.^342^ The CeA, a striatal-like structure, coordinates defensive behaviors and appetite regulation and is extensively connected with other brain regions.^348,349^ Recent studies have shown that somatostatin (SST)⁺ neurons within the CeA selectively encode diverse stimuli and project to the midbrain dopaminergic system, contributing to reward learning. These functional features closely parallel allergen-induced avoidance behaviors.^350^ In experimental colitis models, neurons of the InsCtx were found to encode and retrieve antigen-specific immune responses, acting as a central repository for immune memory. Notably, CeA activation and its projections to the InsCtx were also observed, strongly implicating the CeA as a key node in the formation of allergy-related memory.^85,350^

The CNS can, in turn, modulate local allergic responses. Stress activates the HPA axis, inducing the release of CRH and GCs. CRH promotes mast cell degranulation and proliferation at mucosal sites, whereas GCs can act on GC receptor-expressing ILC2s to shape intestinal inflammation and asthma pathogenesis.^351^ However, the reported effects of GCs are inconsistent, likely reflecting ILC2 heterogeneity.^352–355^ In addition, circadian rhythms regulate allergic responses through oscillatory HPA activity and the expression of clock genes in immune cells, a mechanism that may explain the nocturnal worsening of asthma symptoms.^356–358^ Allergic diseases not only remodel peripheral neural states but may also disrupt neuro-immune homeostasis within the brain. Recent studies have highlighted mucosa-brain interactions and proposed concepts such as the “nose-brain axis”, “lung-brain axis”, and “gut-brain axis”.^359–362^ In allergic rhinitis, elevated nasal mucosal IL-6 may diffuse into the olfactory bulb, leading to hyposmia and implicating anatomical proximity as a potential route for central inflammatory invasion.^363^ Olfactory bulb inflammation has also been linked to depressive phenotypes, and metformin treatment was shown to improve depression-like behaviors by modulating olfactory bulb plasticity through the AMPK/TET2 pathway.^364^ In food-allergic mouse models, autism-like behaviors have been observed, accompanied by altered intestinal serotonin levels and activation of the prefrontal cortex and hypothalamic PVN neurons.^365^ Similarly, milk-allergic mice display reduced social interaction and increased self-grooming, which are correlated with the upregulation of Treg-associated transcription factors in the ileum.^366^ Consistent with these findings, OVA-sensitized mice exhibited activation of the amygdala and PVN, potentially contributing to anxiety-like behaviors.^367^ Together, these observations underscore the profound influence of mucosal immunity on brain function and offer new perspectives for preventing and treating CNS disorders, particularly neuropsychiatric diseases.

#### Autoimmune diseases

In OA, CNS adaptations are further evidenced by monosodium iodoacetate (MIA)-induced models. Brain regions such as the thalamus, hippocampus, and periaqueductal gray exhibited activation patterns linked to pain processing, with similar observations reported in clinical studies (Fig. 4).^368–370^ OA involves a bidirectional brain-bone axis in which brain-derived humoral mediators such as neuropeptide Y (NPY) and vasoactive intestinal peptide (VIP) regulate bone remodeling.^371^ Moreover, bone-derived regulatory molecules can cross the BBB, influencing brain development and cognitive function.^372^ The neuro-immune interactions in OA provide opportunities for targeted therapeutic strategies. Non-invasive brain stimulation techniques such as transcranial magnetic stimulation have been shown to effectively improve OA-induced pain by modulating cortical excitability.^373^ Additionally, targeting humoral pathways of the brain-bone axis, as well as developing drugs that selectively inhibit neuropeptides, neurotransmitters, or their receptors, holds promise for reducing inflammation, slowing cartilage degeneration, and preserving brain function in OA.^374,375^

Neuro-immune interactions also occur within the CNS in RA, where RA exerts widespread effects on neural function. A systematic review proposed that RA may affect inner ear structures, including the cochlea and auditory nerve, leading to hearing impairment.^376^ Furthermore, RA is frequently accompanied by neuropsychiatric comorbidities such as anxiety and neurodegenerative disorders.^377,378^ These comorbidities substantially increase mortality risk in RA patients, with depression accounting for approximately 6.9% of RA-related mortality risk.^379^ Genome-wide association studies (GWAS) have also suggested a genetic correlation between depression and immune-related phenotypes.^380^ Preclinical evidence indicates that chronic inflammatory states such as RA can communicate with the brain through both humoral and neural pathways.^381^ The humoral route primarily involves cytokines such as IL-6, which negatively affect monoaminergic neurotransmission. In the CIA model, elevated IL-1β expression was detected in microglia of the area postrema (AP), a brain region lacking a BBB, and was associated with reduced sucrose preference.^382^ Neural pathways include vagal afferents transmitting peripheral inflammatory signals to the NTS and activating the HPA axis in response to inflammation, thereby modulating emotional and stress responses.^383^ The SNS may also be involved, as RA patients often display autonomic dysfunction characterized by reduced parasympathetic activity and heightened sympathetic tone.^384^ Taken together, these findings suggest that RA profoundly affects CNS function through multilayered neuro-immune dialogs.

#### Immunodeficiency diseases

From the perspective of immune regulation by higher central structures, the brain and related central regions are involved in the integration and modulation of immune information (Fig. 5). Several CNS disorders, such as spinal cord injury (SCI), are reportedly associated with systemic immunosuppression.^216,385,386^ SCI often leads to severe secondary immune suppression, which is referred to as SCI-induced immunodeficiency syndrome (SCI-IDS). The degree of immune deficiency is significantly correlated with the level of injury and the severity of SCI.^387^ One study compared the responses of mice subjected to high thoracic (T3) versus low thoracic (T9) SCI.^388^ Both groups presented elevated serum cortisol levels; however, only the T3 injury group presented a significant increase in splenic NE levels, along with markedly impaired antibody production upon OVA challenge. The administration of a β-adrenergic receptor agonist to T9-injured mice reproduced the SCI-induced immunosuppressive phenotype, further confirming the critical role of ANS dysfunction in this process. Another study demonstrated that splenic sympathectomy in SCI mice successfully eliminated injury-induced immunosuppression.^389^ In addition, upstream mechanisms have been proposed whereby impaired immune function following SCI may be linked to neuroinflammation within the CNS. Neuroinflammation interferes with normal neuronal function, promotes maladaptive plasticity, and induces circuit hyperexcitability. One study revealed that SCI elevates the levels of the proinflammatory cytokine soluble TNF, which triggers immune dysfunction via excessive TNFR1 signaling on excitatory interneurons.^390^ This process was NF-κB-dependent in VGluT2^+^ spinal cord interneurons. The resulting chronic spinal inflammation further altered sympathetic tone to the spleen. Another investigation revealed dysregulation of the neuroendocrine-immune gene network in SCI-IDS.^391^ Specifically, progressive central neuroinflammation post-SCI suppresses hypothalamic Gi-GPCR neuronal signaling and neuropeptide activity within the HPA axis. This further results in the dissociation of autonomic regulation and circadian rhythm between the hypothalamus and the adrenal glands, ultimately leading to hypercortisolism and comprehensive suppression of peripheral adaptive immunity. These findings highlight the central roles of sympathetic nerves, particularly the splenic nerve, and the HPA axis in SCI-IDS. Similarly, stroke has also been shown to induce immunosuppression through analogous mechanisms. Studies have demonstrated that post-stroke, secondary immune organs such as the spleen rapidly undergo atrophy, suggesting pronounced suppression of peripheral immunity.^392^ Furthermore, stroke can activate invariant natural killer T (iNKT) cells in the liver, thereby inducing systemic immunosuppression.^393^ Notably, this process is mediated by NE neurotransmission rather than by specific danger-associated molecular patterns (DAMPs).

Conversely, immunodeficiency can compromise nervous system function by reshaping the immune microenvironment. The characteristics of neurodegenerative diseases may be linked to accelerated aging of the immune system.^394–396^ In patients with acquired immunodeficiency syndrome (AIDS), CNS dysfunction is frequently observed, often manifesting as depressive symptoms, and transcutaneous auricular VNS (taVNS) has been demonstrated to alleviate depression and restore autonomic balance in these patients.^397^ Evidence from studies of neurodegenerative diseases further supports this link. In amyotrophic lateral sclerosis (ALS) models, loss of Tregs accelerates disease progression, while clinical data reveal an inverse correlation between Treg abundance and the rate of disease deterioration.^398^ These findings suggest that endogenous regulatory T cells exert neuroprotective effects beyond their canonical immunoregulatory roles, in part through crosstalk with glial cells. Similarly, deficiency of CD4⁺ T cells has been shown to arrest the maturation of brain-resident microglia at an intermediate stage between fetal and adult phenotypes.^399^ This maturational block results in excessive synapse formation on immature neurons and contributes to aberrant behavioral phenotypes. Together, these studies highlight that immunodeficiency not only weakens host defense but also profoundly influences neural development and function.

## Neuro-immune interactions in infections

**Fig. 6:**
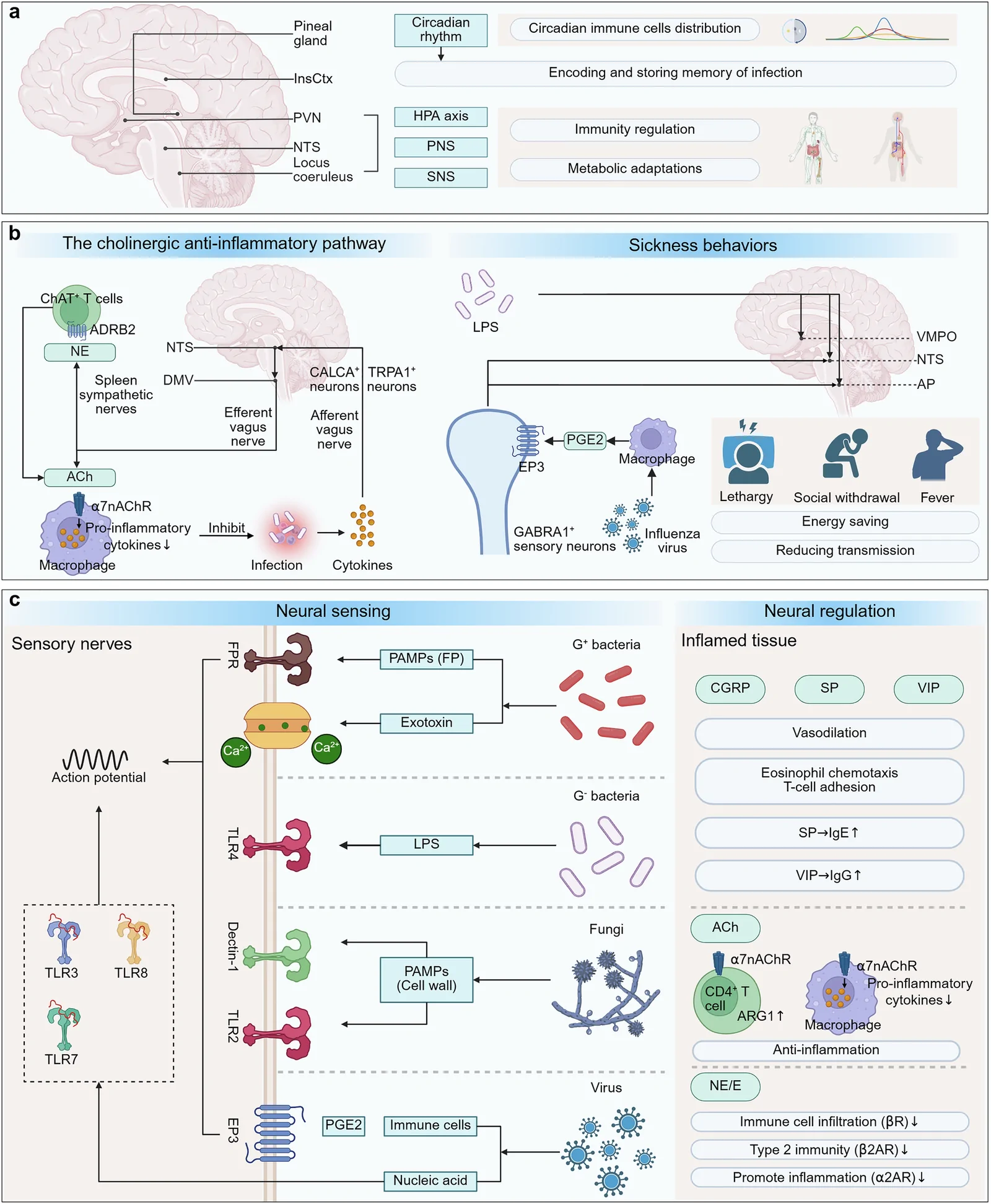
Neuro-immune interactions in infections. Neuro-immune interactions during infection can be categorized into neural sensing and neural regulation. **a** The brain participates in the infection response through central and autonomic pathways. Regions such as the PVN, InsCtx, NTS, and locus coeruleus regulate the HPA axis and autonomic outputs. **b** Neuro-immune reflexes are triggered during infection. The cholinergic anti-inflammatory pathway suppresses systemic inflammation. Simultaneously, reflexes induce behavioral adaptations, such as energy conservation and social withdrawal, to aid host defense. **c** Different pathogens are detected by neurons through specific mechanisms. Upon activation, sensory neurons release neuropeptides such as CGRP and SP to modulate local immune responses, promoting pathogen clearance and influencing antibody production. The figure was created with BioRender.com. ACh acetylcholine, ADCYAP1 adenylate cyclase-activating polypeptide 1, α7nAChR α7 nicotinic acetylcholine receptor, ARG1 arginase-1, AP area postrema, CGRP calcitonin gene-related peptide, DMV dorsal motor nucleus of vagus, EP3 prostaglandin E receptor 3, FP formyl peptide, FPR formyl peptide receptor, HPA hypothalamic-pituitary-adrenal, InsCtx insular cortex, LPS lipopolysaccharides, MAdCAM mucosal vascular addressin cell adhesion molecule-1, NE norepinephrine, NTS nucleus tractus solitarius, PGE2 prostaglandin E2, PVN paraventricular nucleus, PAMP pathogen-associated molecular pattern, SNS sympathetic nervous system, SP substance P, VIP vasoactive intestinal peptide, VMPO ventromedial preoptic nucleus

Neuro-immune interactions orchestrate bidirectional communication in infectious diseases (Fig. 6). On the one hand, peripheral nerves rapidly detect PGE_2_ and relay danger signals to the CNS, triggering sickness behaviors such as fever and anorexia. Conversely, the CNS modulates local immune activity through descending neuroendocrine and autonomic pathways that precisely regulate host defense while preventing excessive immunopathology.^85,400^ This conserved “sense-integrate-respond” circuitry operates across diverse infections, ranging from acute bacterial sepsis to chronic viral persistence. At the local level, neuro-immune interactions participate in sensing infection-related signals and regulating the immune microenvironment at the site of infection. In contrast, the processes of integration and response occur primarily in the brain and its associated neuro-immune reflex pathways, where pathogen-derived information is encoded and multiple physiological systems, including metabolism and motor activity, are coordinated to facilitate host adaptation and mount effective responses to infection.

### Local neuro-immune interactions of infections

Local neuro-immune interactions form the foundation of neuro-immune dialog in infections, involving the direct monitoring and regulation of the immune microenvironment by sensory and autonomic neurons.

#### Neural sensing

Neural sensing of infection occurs through four principal mechanisms: (1) pattern recognition receptor-mediated detection of pathogen-associated molecular patterns through innate immune-like sensing mechanisms, (2) direct activation of neurons by pathogen-derived toxins, (3) pathogen-induced membrane potential alterations modifying neuronal states, and (4) immune cell-derived cytokines, neurotransmitters, and neuropeptides activating neurons post-pathogen recognition.

Pain serves as a hallmark manifestation of neural sensing during infection. Distinct neuro-immune mechanisms have been characterized for major pathogens. *Staphylococcus aureus* employs exotoxins such as α-hemolysin (αHL), leucocidin γ-hemolysin AB, and phenol-soluble modulin α3 (PSMα3) to directly evoke calcium-dependent action potentials in nociceptors.^401,402^ Similarly, *Streptococcus pyogenes* utilizes streptolysin S (SLS) exotoxin to trigger neuronal depolarization.^403^ Gram-positive bacteria primarily produce exotoxins that modulate membrane potential, although they also engage canonical pattern recognition pathways. For instance, *Staphylococcus aureus*-derived N-formylated peptides activate formyl peptide receptors (FPRs), a class of pattern recognition receptors (PRRs), on sensory neurons, altering membrane potentials and potentiating pain signaling.^401^ In contrast, gram-negative pathogens employ divergent strategies. The endotoxin LPS binds TLR4 receptors expressed on sensory neurons.^404,405^ LPS-mediated TLR4 activation in sensory neurons enhances TRPV1 and TRPA1 sensitivity and stimulates CGRP release.^406–408^ Intriguingly, LPS can also directly modulate neuronal activity through TRPV4 ion channel activation independent of TLR4 signaling.^409^ This evidence suggests the sophisticated integration of pattern recognition and ion channel modulation in infectious neuro-immune crosstalk.

Fungal pathogens employ analogous neuro-immune activation strategies. Sensory neurons detect fungal cell wall components through PRRs, including TLR2 and Dectin-1. Exposure to zymosan or heat-killed *Candida albicans* triggers immediate calcium influx and CGRP release.^410–412^ Viral detection primarily occurs through intracellular PRRs (TLR3, TLR7, TLR8) that detect pathogen-derived nucleic acids. Activation of these receptors in neurons triggers neuronal dendritic retraction, potentially altering local neuro-immune communication.^413^ In addition to classical pattern recognition, influenza-induced PGE_2_ release activates GABRA1^+^ glossopharyngeal neurons, transmitting infection signals to the PBN to coordinate systemic sickness behaviors.^400^

Neuronal plasticity represents a fundamental feature of neuro-immune interactions. Clinically, chronic pelvic pain after urinary tract infections, even after antibiotic treatment, correlates with NGF-mediated axonal sprouting driven by bladder-resident immune cells. This persistent structural remodeling of sensory neurons may represent a form of neuron-based “immune memory”.^62^ Molecular convergence underlies functional synergy. Neurons and immune cells utilize overlapping components: PRRs, neurotransmitters, neuropeptides, and cytokines. This evolutionary strategy provides dual advantages: it reduces the metabolic costs of developing parallel systems while enabling cross-system synergy. Immune cells decode physiological stress signals through neurotransmitter receptors, while neurons monitor the microenvironmental status via cytokine signaling. Together, these shared pathways constitute an integrated sensory defense network that coordinates organism-wide responses to challenges.

#### Neural regulation

At the local level, sensory and autonomic nerves are pivotal for neural regulation. Sensory neurons not only detect threats but also modulate local immune states by releasing neuropeptides, neurotransmitters, and, in some cases, certain cytokines or chemokines. A classic example is neurogenic inflammation, wherein infection-induced neuropeptides such as CGRP and SP promote vasodilation and edema, thereby facilitating immune cell recruitment and pathogen clearance.^414^ These neuropeptides further shape immune microenvironments, similar to their roles in tumors and immune-related disorders. CGRP has been shown to establish an immunosuppressive microenvironment in certain diseases.^47,111,240^ Conversely, it can also exert proinflammatory effects, including eosinophil chemoattraction and T-cell adhesion.^415,416^ Furthermore, recent studies have revealed that CGRP induces an anti-viral Th1 cell response via the RAMP3 downstream pathway during acute viral infections. Notably, the dual functions of CGRP are not mutually exclusive. It may reflect its ability to modulate diverse immune cell populations, including T cells at various differentiation states along the effector-to-exhausted continuum.^417,418^

In murine models of *Streptococcus pneumoniae* infection and *Alternaria*-induced asthma, sensory neurons exhibit stimulus-dependent neuropeptide release. VIP is more prominently involved in bacterial infection, whereas SP is preferentially secreted in allergic asthma.^419^ Functionally, VIP enhances IgG production by B cells, while SP drives IgE release. This suggests that sensory neuron-derived neuropeptides inhibit infections but may exacerbate allergic responses, potentially influencing allergic inflammation. Although the precise mechanistic basis for this stimulus-specific neuropeptide release remains unresolved, the data imply the existence of an intrinsic neuronal program that tailors immune responses to specific threats.

The ANS can orchestrate local immune responses. Traditionally considered to act primarily through efferent neurotransmitter signaling, emerging evidence reveals substantial afferent contributions. In the parasympathetic branch, activation of the vagus nerve-α7nAChR pathway in post-influenza lungs upregulates arginase-1 (ARG1), mediating anti-inflammatory and tissue-protective effects.^420^ Conversely, in bacterial pneumonia models, vagal afferents release CGRP, suppressing neutrophil recruitment and γδ T-cell function and ultimately impairing bacterial clearance.^421^ Influenza A-induced pneumonia studies have revealed that inhibiting vagal afferent activity reduces lung inflammation but exacerbates neuroinflammation and clinical severity.^422^ This likely reflects the dual roles of the vagus nerve in local immune modulation and systemic anti-inflammatory reflexes mediated by splenic efferent nerves. Similarly, sympathetic nerves exhibit context-dependent immunomodulation. Optogenetic activation of colonic sympathetic fibers upregulates mucosal addressin cell adhesion molecule-1 (MAdCAM-1) on endothelial cells, ameliorating colitis by reducing immune cell infiltration.^423^ In contrast, systemic sympathetic activation via the sympathetic-adrenal axis promotes proinflammatory “alert” responses. *Clostridioides difficile* colitis models reveal further complexity. Adrenergic nerves suppress β2AR^+^ ILC2s to limit type 2 immunity, whereas colonic sympathetic NE exacerbates inflammation via α2AR, a receptor typically associated with sympathetic inhibition. This paradoxical finding implies that α2AR may act as a postsynaptic heteroreceptor, although further validation is needed.^57,424^

Overall, sensory and autonomic nerves shape immune responses in a highly context-dependent manner. Through distinct mediators such as CGRP, VIP, and adrenergic neurotransmitters, they can either suppress or promote inflammation, reflecting an intrinsic flexibility that enables the nervous system to fine-tune immunity across infections, allergies, and tissue homeostasis.

### Neuro-immune reflexes in infections

This section addresses two major topics, including the role of the anti-inflammatory reflex during infection and the mechanisms and physiological significance of sickness behaviors. In the LPS-induced endotoxemia model, peripheral inflammatory signals are encoded by sensory neurons of distinct functional subtypes within the vagal ganglia.^74^ Specifically, TRPA1⁺ neurons predominantly sense anti-inflammatory cytokines such as IL-10, whereas CALCA⁺ neurons preferentially detect proinflammatory cytokines such as IL-1β, IL-6, and TNF. These signals converge in the NTS, where DBH⁺ glutamatergic neurons constitute a central integrative hub, thereby forming the afferent arm of the anti-inflammatory reflex. As described above, the efferent arm of the vagus nerve either directly releases ACh in peripheral tissues or, via the splenic sympathetic nerve, releases NE to activate splenic CD4⁺ChAT⁺ T cells, which subsequently secrete ACh. Ultimately, ACh binds to α7nAChR on macrophages, suppressing the release of proinflammatory cytokines.^71,425–427^ Some studies have demonstrated that electrical stimulation of the efferent vagus nerve suppresses the proinflammatory cytokine cascade in endotoxemia, thereby limiting the spread of systemic inflammation and improving survival in endotoxemic animal models, and, in contrast, vagotomy has opposite effects.^425,428^ A clinical trial evaluated the effects of taVNS in patients with sepsis, with TNF-α levels as the primary endpoint.^429^ This study suggested that taVNS may reduce proinflammatory cytokines while enhancing anti-inflammatory cytokines. However, another study reported that prolonged VNS led to increased germinal center apoptosis and impaired plasma cell differentiation in mouse models.^430^ Given that insufficient humoral immune responses are associated with poor outcomes in sepsis patients, the finding that chronic VNS can impair humoral immunity in animal models suggests that prolonged VNS might carry a risk of excessive immunosuppression in sepsis. This underscores the need for cautious design of clinical VNS interventions.^431^

Sickness behaviors, characterized by lethargy, anorexia, and social withdrawal, facilitate host adaptation to infection, increase disease resistance, and restrict pathogen transmission.^432^ Recent studies have begun to elucidate the neuro-immune mechanisms underlying their induction. For instance, during influenza virus infection, glossopharyngeal GABRA1⁺ neurons sense excessive local production of PGE_2_ and project to the lateral NTS, relaying signals to the brain to elicit behavioral responses.^400^ In LPS-induced bacterial infection models, ADCYAP1⁺ neuronal populations in the brainstem NTS and AP are markedly activated.^433^ Additional work in LPS models has identified the organum vasculosum of the lamina terminalis (OVLT) and the adjacent ventromedial preoptic area (VMPO) in the hypothalamus as key regions regulating core body temperature, thermogenic behavior, and appetite.^434^ Collectively, these findings highlight the evolutionary conservation of the NTS and hypothalamic circuits in mediating sickness behaviors across diverse infections. Nevertheless, how these regions coordinate with other brain areas to ultimately shape behavioral adaptations remains an open question for future research.

### Brain processing immune information about infections

Upon receiving immune-related signals in regions such as the InsCtx, the brain integrates this information through complex neural and humoral mechanisms, subsequently coordinating physiological functions and behavioral responses to ensure that systemic activities align with the demands of the immune system in defending against pathogenic insults.^20^

The CNS coordinates neuro-immune interactions via systemic regulatory mechanisms. Higher-order brain areas, alongside the brainstem and hypothalamic nuclei, constitute the central autonomic network, which is critical for sustaining homeostasis through dynamic autonomic regulation.^435–438^ The CNS integrates diverse neural, endocrine, and immune signals to coordinate local and systemic immunity during infection. For instance, InsCtx encodes and retains memory of gut inflammation; reactivating these neurons post-resolution triggers colitis relapse in dextran sulfate sodium (DSS)-induced models, suggesting analogous mechanisms may operate during infections.^85^ While the immune system generates pathogen-specific responses, whether the CNS discriminates immune microenvironments to encode pathogen-specific memory remains unexplored. Recent work investigating infection-associated immune memory has focused on microglia, the resident immune cells of the brain.^439^ In a repeated low-dose LPS model, microglia displayed dynamic functional shifts from immune activation to tolerance. Interestingly, microglia also experienced morphological amoeboid-to-hypertrophic transformation with end-to-end connections.^439^ LPS-induced microglial activation may involve meningeal/endothelial TLR4, as LPS penetrates the BBB significantly only at high doses, while these morphological alterations correlate with neurological disorders, underscoring the adverse neural impacts of infections. This work posits direct immune signal perception by CNS immune cells and raises clinically pertinent questions about neuro-immune crosstalk in neurological diseases, such as how neural pathologies might modulate immune responses in infections.

Moreover, the CNS also coordinates multiple physiological systems to collectively resist pathogen invasion and restrain inflammatory responses. During acute infection, hypothalamic nuclei involved in feeding and metabolism, such as the arcuate nucleus and the lateral hypothalamic area, are activated, underscoring the role of the brain in integrating metabolism, behavior, and immune responses.^440^ Acute infections are frequently accompanied by metabolic reprogramming, in which the HPA axis serves as a critical mechanism for metabolic adaptation.^441^ Proinflammatory cytokines such as TNF-α released by immune cells activate the PVN and hypothalamus, thereby driving HPA axis activation and GC release. GCs, in turn, suppress excessive immune activation, forming a neuro-immune feedback loop.^442^

In addition to being involved in neuroendocrine regulation, the SNS innervates multiple metabolic organs and contributes to metabolic adaptation during infection. For instance, the hypothalamus-SNS-liver axis promotes stress-induced hyperglycemia, which may help meet the heightened energy demands of the immune system during acute infection.^443,444^ Circadian rhythms, as central regulators of diverse physiological processes, also exert profound effects on immune function. HSC trafficking from the bone marrow to the blood follows robust circadian oscillations.^445–447^ Nighttime LPS challenge significantly reduced survival in mice compared to daytime challenge, highlighting the critical role of circadian rhythms in shaping immune responses.^447^ Sleep, as a manifestation of circadian regulation, reduces energy expenditure by lowering heart rate and body temperature, thereby supporting recovery from infection and enhancing immune competence.^448^ Recent studies further demonstrated that, after myocardial infarction, monocyte infiltration into the thalamic posterior nucleus enhances slow-wave sleep through TNF signaling, which suppresses cardiac sympathetic output, attenuates inflammation, and promotes cardiac repair.^199^ Similarly, brainstem ADCYAP1⁺ neuronal populations respond to peripheral infection by reducing activity and inducing sickness behavior, thereby facilitating host defense.^433^ Moreover, InsCtx functions as an important hub for encoding and storing immune-related information, while sleep has been shown to enhance immune memory in a manner analogous to its role in learning and memory consolidation.^85,449,450^

In summary, the CNS enhances host adaptation to infection and strengthens immune defense by orchestrating multisystem mechanisms, including neuroendocrine regulation, autonomic control, and circadian modulation.

## Targeted therapies

### Trends of clinical trials

Our systematic analysis of clinical trial registries identified over 8000 registered trials investigating neurological interventions since 1984. These therapeutic approaches fall into four main categories, including surgical, pharmacological, neuromodulation, and other emerging modalities. The evolution of these interventions over four decades reveals three key paradigm shifts. First, surgical neuro-targeting has transitioned from invasive procedures to minimally invasive techniques. Second, pharmacological interventions have progressed from classical receptor blockers to biologics such as NGF and the repurposing of established drugs. Third, neuromodulation technologies have advanced from physical/mechanical approaches to modern implantable and noninvasive devices utilizing electromagnetic stimulation paradigms, now applied in chronic pain and epilepsy.^451,452^ Recent years have witnessed transformative breakthroughs, including landmark clinical trials of brain-computer interfaces and the rapid translation of novel biotechnologies such as adeno-associated virus-mediated NGF gene therapy for neural regeneration. This trajectory demonstrates both continuous innovation in neurological interventions and the expanding clinical applications of conventional therapeutic modalities.

Our systematic analysis identified 91 active clinical trials (24 oncological, 67 non-oncological) investigating neuro-immune interactions through primary endpoints or study objectives, excluding terminated or suspended trials (Table 1). The majority of oncology trials, predominantly phase II, with over 50% initiated within the past 5 years, have focused on breast, genitourinary, and gastrointestinal cancers. These tumors are characterized by dense innervation. Therapeutic approaches in oncology remain relatively limited, primarily exploring β-adrenergic blockade combined with standard therapies and perioperative neural blockade techniques. In contrast, non-oncological trials have focused primarily on immune-mediated diseases, including MS, RA, SLE, and asthma, with emerging investigations in patients with COVID-19, stroke, and depression. Compared with oncology trials, non-oncological studies have employed a broader range of interventions, most notably VNS and transcutaneous electrical nerve stimulation.

**Table 1:** Clinical trials targeting neuro-immune interactions

Classification	Intervention	ID	Phase	Conditions	Status	Objective
Tumor	Lidocaine	NCT06703437	1	Prostate cancer	Not recruiting	Efficacy and safety
Tumor	Propranolol	ACTRN12615000889550	2	Breast cancer	Completed	Gene expression profile
Tumor	Morphine	NCT04689945	Not applicable^a^	Breast cancer	Completed	Effect on inflammatory and immune responses
Tumor	Chemotherapy, propranolol	NCT01504126	1	Ovarian cancer	Completed	Efficacy
Tumor	Propranolol	NCT01308944	1	Ovarian cancer	Completed	Efficacy
Tumor	Propranolol	NCT02013492	1	Breast cancer, melanoma, ovarian cancer	Completed	Efficacy
Tumor	Fentanyl, β-blocker (esmolol, metoprolol)	NCT01544959	Not provided	Breast cancer	Completed	Postoperative pain and prognosis
Tumor	Propranolol, etodolac	NCT00502684	Not provided	Breast cancer	Completed	Cellular immunity and cancer recurrence rates
Tumor	Propranolol	NCT05679193	2	Prostate cancer	Completed	Efficacy
Tumor	Propranolol, camrelizumab	ChiCTR2100047864	2	Intrahepatic cholangiocarcinoma	Not recruiting	Efficacy and safety
Tumor	VNS	NCT06817161	3	Ovarian cancer	Recruiting	Quality of life
Tumor	Propranolol, naltrexone	NCT05968690	1	Melanoma	Recruiting	Efficacy and safety
Tumor	Surgery, propranolol, parecoxib	ChiCTR-IPR-14005271	1	Breast cancer	Recruiting	Effects on regulatory T-cell frequency and function
Tumor	Fluorouracil, leucovorin, oxaliplatin, pembrolizumab, propranolol	NCT05651594	2	Esophageal/Esophagogastric junction adenocarcinoma	Recruiting	Efficacy
Tumor	Propranolol, etodolac	NCT03919461	2	Colorectal cancer	Recruiting	Efficacy
Tumor	Pembrolizumab, propranolol, nivolumab, avelumab	NCT04848519	2	Urothelial cancer	Recruiting	Efficacy and safety
Tumor	Propranolol, sintilimab, chemotherapy	NCT05979818	1	Non-small cell lung cancer	Recruiting	Efficacy and safety
Tumor	Durvalumab, gemcitabine, nab-paclitaxel, tremelimumab, propranolol, cisplatin	NCT05451043	2	Pancreatic cancer, hepatocellular cancer, biliary tract cancer, cholangiocarcinoma	Recruiting	Efficacy
Tumor	Propranolol	NCT06145074	2	Pancreatic cancer	Recruiting	Efficacy
Tumor	Propranolol, etodolac	NCT03838029	2	Pancreatic cancer	Recruiting	Efficacy and safety
Tumor	Propranolol, etodolac	NCT00888797	3	Colorectal cancer	Last known status was: Recruiting (2011-07)	Efficacy
Tumor	Sorafenib, VT-122 (propranolol plus etodolac)	NCT01265576	2	Hepatocellular cancer	Last known status was: Active, not recruiting (2015-12)	Efficacy and safety
Tumor	Spinal anesthesia, general anesthesia	NCT01588847	Not provided	Melanoma	Last known status was: Recruiting (2014-12)	The long-term outcomes, impact on perioperative immunoediting, and new potential predictive biomarkers
Tumor	Metoprolol, infliximab, etanercept, tocilizumab	NCT04082910	1/2	Solid tumor, hematological malignancy	Last known status was: Recruiting (2022-04)	Efficacy and effects on the serum levels of IL-6 and other cytokines
Immune-related disorders	VNS	NCT05007743	Not provided	Restless legs syndrome	Active not recruiting	Efficacy and safety
Immune-related disorders	tENS	NCT05230628	Not provided	Obesity	Active not recruiting	Efficacy
Immune-related disorders	Neurostimulation of the stellate ganglion	NCT01724307	Not applicable^b^	Asthma	Completed	Efficacy
Immune-related disorders	tENS	NCT00824642	Not applicable^b^	Asthma	Completed	Efficacy
Immune-related disorders	Glatiramer acetate, albuterol	NCT00039988	Not applicable^b^	MS	Completed	Efficacy
Infection	Auricular percutaneous neurostimulation	NCT04514627	Not applicable^b^	COVID-19	Completed	Efficacy
Immune-related disorders	Clonidine	NCT02268448	1	Chronic itch in atopic dermatitis	Completed	Efficacy and safety
Immune-related disorders	VNS	NCT01569789	1	Healthy adults	Completed	Effects on the serum levels of inflammatory cytokines
Immune-related disorders	Slow deep breathing	NCT04253444	Not applicable^b^	Gastroesophageal reflux	Completed	Efficacy
Infection	Pyridostigmine	NCT02850276	1	HIV disease	Completed (Published at clinicaltrials.gov)	Assessing vagus nerve dysfunction in HIV infection
Immune-related disorders	Lipopolysaccharide	NCT01352871	Not applicable^a^	Innate immune response	Completed	Effects on the innate immune response
Immune-related disorders	Genicular nerve block, intra-articular steroid injection	NCT04687930	4	Arthritis	Completed	Efficacy
Immune-related disorders	Portable neuromodulation stimulator	NCT04498039	Not applicable^b^	MS	Completed	Efficacy
Immune-related disorders	tES	NCT02019927	Not applicable^b^	MS	Completed	Efficacy
Immune-related disorders	Phenytoin	NCT01451593	2	Optic neuritis, MS	Completed	Efficacy
Immune-related disorders	tDCS and EAC sham	NCT01747070	Not applicable^b^	OA	Completed	Efficacy
Immune-related disorders	Nerve block	NCT04130178	Not applicable^b^	RA	Completed	Efficacy
Immune-related disorders	VNS	NCT03437473	Not applicable^b^	RA	Completed	Efficacy and safety
Immune-related disorders	Cyberonics VNS system	NCT01552941	Not applicable^b^	RA	Completed	Efficacy and safety
Immune-related disorders	Radiofrequency	NCT03852355	Not applicable^b^	RA	Completed	Efficacy
Immune-related disorders	Stellate nerve block	NCT05932485	Not applicable^b^	Atrial fibrillation	Completed	Efficacy
Immune-related disorders	Carbamazepine	NCT04371575	Not applicable^a^	Trigeminal neuralgia, MS	Completed	Efficacy
Immune-related disorders	Phenylephrine, 25 micro-Ci of radiation	NCT02725060	Not applicable^b^	Cardiovascular diseases	Enrolling	Efficacy
Immune-related disorders	VNS	NCT06389175	Not applicable^b^	Depressive disorder	Enrolling	Efficacy
Immune-related disorders	VNS	NCT06779123	Not applicable^b^	RA	Enrolling	Efficacy and safety
Immune-related disorders	Measurement of blood immune parameters	NCT04211636	Not applicable^b^	Spinal cord trauma	Not yet recruiting	Effects on the immune function
Immune-related disorders	TAP Block	NCT05840406	Not applicable^b^	Postoperative pain	Completed	Efficacy
Immune-related disorders	tENS	NCT06432686	Not applicable^b^	MS	Not yet recruiting	Efficacy
Immune-related disorders	rTMS and percutaneous tibial nerve stimulation	NCT06581341	Not applicable^b^	Neurogenic overactive bladder, MS	Not yet recruiting	Efficacy
Immune-related disorders	Acupuncture, VNS	NCT06875102	Not applicable^b^	Sjogren syndrome	Not yet recruiting	Efficacy
Immune-related disorders	Posterior nasal nerve neurectomy	NCT06887842	Not applicable^b^	Intractable allergic rhinitis	Enrolling by invitation	Efficacy
Immune-related disorders	VNS	NCT03953768	Not applicable^b^	Autoimmune diseases	Recruiting	Efficacy
Immune-related disorders	Stimulating the splenic neurovascular bundle	NCT05003310	Not applicable^b^	RA	Recruiting	Efficacy
Immune-related disorders	VNS	NCT05421208	Not applicable^b^	Long COVID-19	Recruiting	Effects on autonomic nervous function
Immune-related disorders	tENS	NCT05500963	Not applicable^b^	MS	Recruiting	Efficacy
Immune-related disorders	VNS	NCT05588063	Not applicable^b^	Nephrotic syndrome	Recruiting	Efficacy
Immune-related disorders	Parasym 1 Hz/30 Hz	NCT05704153	Not applicable^b^	SLE	Recruiting	Efficacy
Immune-related disorders	VNS	NCT05730049	Not applicable^b^	Spinal cord injuries	Recruiting	Efficacy
Immune-related disorders	Music therapy	NCT05777499	Not applicable^b^	Autoimmune diseases	Recruiting	Efficacy
Immune-related disorders	TNS, parasacral nerve stimulation	NCT06143397	Not applicable^b^	MS	Completed	Efficacy
Immune-related disorders	VNS	NCT06527547	Not applicable^b^	RA	Recruiting	Efficacy
Immune-related disorders	Electroacupuncture	NCT06643247	Not applicable^b^	Herpes zoster	Recruiting	Efficacy
Immune-related disorders	tENS	NCT02917265	2	SLE	Terminated	Efficacy
Immune-related disorders	Hypoglossal nerve stimulation treatment withdrawal	NCT03051165	Not applicable^b^	Obstructive sleep apnea	Terminated	Efficacy
Immune-related disorders	Non-invasive VNS	NCT03772717	Not applicable^b^	Demyelinating polyneuropathy	Terminated	Efficacy
Immune-related disorders	VNS	NCT04256837	Not applicable^b^	Kidney transplant recipients	Terminated	Effects on cytokines
Immune-related disorders	Probiotic	NCT00418340	4	Irritable bowel syndrome	Last known status was: Not yet Recruiting (2007-11)	Effects on visceral hypersensitivity
Immune-related disorders	Saliwell crown	NCT01174329	2/3	Sjögren’s syndrome	Last known status was: Recruiting (2010-07)	Efficacy
Immune-related disorders	VNS	NCT01378611	2/3	Refractory epilepsy in children	Last known status was: Active, not recruiting (2011-06)	Efficacy
Immune-related disorders	Cyberonics VNS system	NCT01552538	1/2	RA	Last known status was: Active, not recruiting (2016-10)	Efficacy
Immune-related disorders	Antipyrine-benzocaine otic solution	NCT02153541	2	Asthma	Last known status was: Not yet recruiting (2023-02)	Efficacy
Immune-related disorders	General anesthesia induction	NCT02154074	4	Elderly diabetes	Last known status was: Not yet recruiting (2014-05)	Effects on perioperative stress response and immune function
Immune-related disorders	VNS	NCT02620176	Not applicable^b^	Esophageal diseases	Last known status was: Recruiting (2015-11)	Efficacy
Immune-related disorders	Photobiomodulation in the sublingual region	NCT03360487	2	MS	Completed	Effects on IL-10 and nitrites
Immune-related disorders	tENS	NCT04042363	Not applicable^b^	MS, Optic neuritis	Last known status was: Recruiting (2019-07)	Effects on myelin repair
Immune-related disorders	Minocycline	NCT04478500	3	Resistant hypertension	Last known status was: Recruiting (2022-09)	Efficacy
Immune-related disorders	No innervation	NCT04550884	Not applicable^b^	RA	Last known status was: Not yet recruiting (2020-09)	Evaluating neuropathy using nerve conduction studies and neuromuscular ultrasound
Immune-related disorders	Sacral nerve stimulation	NCT04821050	Not applicable^b^	RA	Last known status was: Recruiting (2021-03)	Efficacy
Immune-related disorders	VNS	NCT04862117	Not applicable^b^	RA	Active, not recruiting	Efficacy and safety
Immune-related disorders	tENS	NCT05115565	Not applicable^b^	Total knee replacement	Last known status was: Not yet recruiting (2021-10)	Efficacy
Immune-related disorders	Local anesthetic/steroid mixture	NCT05292677	4	Chronic cough	Last known status was: Recruiting (2022-03)	Efficacy
Immune-related disorders	rTMS	NCT05312138	Not applicable^b^	Neurogenic bladder dysfunction	Completed	Efficacy
Immune-related disorders	VNS	NCT05315739	Not applicable^b^	SLE	Completed	Efficacy
Immune-related disorders	VNS	NCT05434403	Not applicable^b^	CIPN	Last known status was: Enrolling by invitation (2022-06)	Efficacy
Immune-related disorders	Pentoxifylline	NCT03859570	4	Lupus nephritis	Withdrawn	Efficacy
Immune-related disorders	Intranasal neurostimulator	NCT04240587	Not applicable^b^	Contact allergy	Withdrawn	Efficacy
Infection	VNS	NCT04774705	Not applicable^b^	Sepsis	Recruiting	Efficacy

*CIPN* chemotherapy-induced peripheral neuropathy, *MS* multiple sclerosis, *OA* osteoarthritis, *RA* rheumatoid arthritis, *rTMS* repetitive transcranial magnetic stimulation, *SLE* systemic lupus erythematosus, *ENS* electrical nerve stimulation, *tES* transcorneal electrical stimulation, *TNS* tibial nerve stimulation, *VNS* vagus nerve stimulation

^a^Observational clinical trials

^b^Interventional studies involving devices or behaviors, or studies that do not involve new drugs

Three distinct patterns emerge from the clinical translation of neuro-immune research: (1) pronounced interdisciplinary integration, juxtaposing neuromodulation technologies with biological agent-based approaches; (2) polarization of the disease spectrum, characterized by innervation-rich solid tumors on one end and immune-mediated chronic inflammatory conditions on the other; and (3) asymmetrical developmental progress, with oncological applications largely in phase II clinical validation, contrasting with established non-oncological therapeutic uses.

### Clinical evidence supporting efficacy

The therapeutic efficacy of neuro-immune interventions demonstrates both significant promise and persistent challenges across diverse disease contexts. Within oncology, clinical investigation has focused predominantly on β-adrenergic receptor blockade strategies (Table 1). A phase II randomized controlled trial (ACTRN12615000889550) involving 60 patients assessed the effects of preoperative propranolol-induced β-blockade on biomarkers of metastatic potential and immune cell profiles within primary breast tumors. The study revealed that one week of propranolol treatment reduced intratumoral mesenchymal polarization and enhanced immune cell infiltration in early-stage, surgically resected breast cancer. Another perioperative clinical trial (ChiCTR-IPR-14005271), which enrolled 101 breast cancer patients, demonstrated that propranolol administration attenuated stress-induced expansion of peripheral blood Tregs.^453^ Complementary in vitro experiments using peripheral blood mononuclear cells from breast cancer patients confirmed that epinephrine significantly promoted Treg proliferation. Additionally, a separate perioperative study in patients with breast cancer evaluated the combined effects of a cyclooxygenase-2 inhibitor (etodolac) and propranolol, conducting transcriptomic analysis on surgical specimens.^454^ The results demonstrated that pharmacological intervention significantly attenuated epithelial-mesenchymal transition, suppressed pro-metastatic and proinflammatory transcription factors, reduced tumor-infiltrating monocytes, and increased the number of tumor-infiltrating B cells. The treatment also abrogated preoperative elevations in serum IL-6 and CRP levels, prevented surgery-induced declines in IL-12 and IFN-γ production, and increased CD11a expression on circulating NK cells (NCT00502684).^455^ Similar immunological effects were observed in colorectal cancer clinical trials, suggesting conserved neuro-immune mechanisms across cancer types (NCT00888797).^456^ Several ongoing clinical trials are investigating combined immunotherapy and propranolol, although results remain pending (NCT04848519, ChiCTR2100047864, and NCT05968690). Notably, β-blockers exhibit therapeutic potential in mitigating treatment-related adverse effects. For instance, one study evaluated metoprolol for preventing cytokine release syndrome in CAR-T therapy patients (NCT04082910). Although targeted sensory nerve therapy has not yet been applied in clinical practice, preclinical studies investigating the interaction between sensory neurons and immune cells in tumors have yielded promising results. Hyperthermia serves as an adjuvant therapy for tumors, and the TRPV1 channel is activated at temperatures ≥43°C.^457,458^ In vivo studies have demonstrated that mild photothermal-assisted immunotherapy synergistically enhances CD8^+^ T-cell infiltration into the TME and improves sensitivity to immunotherapy in 4T1 and B16F10 murine models.^459^ This finding was corroborated by a separate study in which capsaicin-mediated TRPV1 activation upregulated PD-L1 in tumor cells and promoted CD8^+^ T-cell recruitment.^460^ Additional studies have indicated that TRPV1 blockade suppresses heat-induced calcium influx and subsequent HSF1 nuclear translocation, thereby potentiating hyperthermia efficacy across primary, metastatic, and recurrent tumor models.^461^

In non-oncological diseases, neuromodulation has emerged as a promising therapeutic approach because of its non-invasive nature (Table 1). A two-phase multicenter clinical trial evaluated a novel miniaturized VNS device for drug-refractory RA. The trial demonstrated significant symptom improvement with a relative safety profile (NCT03437473).^462^ Another RA trial encompassing mild, moderate, and refractory cases investigated sacral nerve stimulation, an alternative parasympathetic modulation strategy, although the results remain unpublished (NCT04821050). A study assessing transcutaneous cymba concha vibrotactile stimulation (targeting auricular vagal branches) reported 80%, 73%, and 50% reductions in serum TNF-α, IL-6, and IL-1β levels, respectively, in healthy subjects, with anti-inflammatory effects persisting for 24 h. RA patients maintained significant reductions in disease activity seven days post-treatment (NCT01569789, NCT00859859).^463^ These effects are likely mediated through vagally regulated anti-inflammatory reflex pathways. Intriguingly, while vagal signaling typically enhances gastrointestinal function and has been associated with peptic ulcer development, an ongoing trial is exploring its transcutaneous application for esophageal pain relief (NCT02620176).^464^ This suggests potential functional antagonism between systemic anti-inflammatory effects and local physiological actions, or alternatively, spatiotemporal heterogeneity in vagal-immune interactions. Another study evaluated non-invasive tongue electrostimulation in MS patients with gait impairment: among 20 participants, combined electrostimulation-exercise therapy outperformed exercise alone in improving mobility without significant adverse events (NCT04498039). Collectively, these findings demonstrate that non-invasive neuromodulation offers cost-effective therapeutic potential across diverse immune-related disorders.

### Factors limiting therapeutic efficacy

#### Spatiotemporal heterogeneity as a clinical challenge

A previous study proposed a dynamic model describing the spatiotemporal heterogeneity of neuro-immune interactions, and this spatiotemporal heterogeneity also poses a challenge for clinical translation. Emerging evidence suggests cancer-type specificity in adrenergic-immune interactions. SNS activity may paradoxically inhibit tumor progression in pancreatic cancer models, although confirmatory clinical data from ongoing trials such as NCT05451043 remain pending. This observation indicates a critical knowledge gap in our understanding of neuro-immune crosstalk in oncology. The spatiotemporal heterogeneity of these interactions, varying by tumor type, anatomical location, and disease stage, currently represents a fundamental challenge for developing effective targeted neuro-immunotherapies. Short-term VNS has been shown to alleviate inflammation and pain in immune-related disorders such as RA, OA, and SLE. However, some studies report that prolonged stimulation may suppress humoral immune responses.^430^ Addressing these spatiotemporal dynamics will be essential for the design of targeted neuro-immune therapies.

Bridging this gap requires coordinated efforts in both basic and clinical research. In basic research, high-resolution maps of neuro-immune interactions should be constructed, with spatial transcriptomics and single-cell omics serving as powerful tools to achieve this goal. Clinically, systematic sample collection from patient cohorts is essential to comprehensively assess the effects of therapeutic interventions on immune and neural functions, thereby enabling precise patient stratification.

#### Managing off-target effects in neuro-immunomodulation

The nervous system represents the central regulatory network of the human body, with evidence of neural regulation documented in nearly all tissues. Consequently, therapeutic strategies targeting neural pathways, including pharmacological interventions, neuromodulation, and surgical procedures, often exert systemic side effects on other physiological systems.^2,465^

In the perioperative treatment of early-stage breast cancer, the administration of β-blockers not only reduces the immunosuppressive function of stromal cells within tumors after surgery but also enhances immune cell infiltration into the TME. This intervention is accompanied by a decreased heart rate and blood pressure. Interestingly, patients who exhibited such pharmacodynamic responses, manifested by reductions in heart rate and blood pressure, also presented significantly increased intratumoral infiltration of CD68⁺ macrophages and CD8⁺ T cells, suggesting that the immunological effects of β-blockers may be closely linked to their systemic pharmacological actions.^11^ Current neural-immune targeting strategies in oncology are primarily drug-based. In contrast, for immune-related disorders, neuromodulation technology has been extensively explored as a novel therapeutic avenue. However, a significant challenge for its clinical application is the precise targeting of specific neuro-immune mechanisms.^466,467^ For example, in clinical trials, patients receiving VNS frequently report adverse effects such as cough, sore throat, and dysphonia, which may arise from off-target stimulation of non-cardiac vagal branches.^468^

Off-target effects underscore the need for more precise intervention strategies. From a basic research perspective, it is essential to comprehensively map the tissue-specific projections of neural circuits.^467^ Recent studies integrating single-cell transcriptomics, neural tracing, and three-dimensional imaging have generated high-resolution atlases of neural regulation at the single-cell level, providing a valuable resource for precision therapy.^469^ From a clinical standpoint, establishing dedicated neuro-immune treatment cohorts is crucial for systematically analyzing the relationships between baseline patient characteristics, such as genotype, drug metabolism capacity, immune status, and therapeutic responses, thereby facilitating optimization of individualized treatment regimens. For pharmacological interventions, advances in drug delivery methods, such as precise targeting of pathological sites, are needed.^470^ For neuromodulation interventions, further refinement of stimulation parameters, including frequency and intensity, will be necessary to maximize efficacy while minimizing the risk of off-target effects.

## Challenges and future directions of neuro-immune research

### Outstanding issues in neuro-immune research

The interplay between the nervous and immune systems has long been recognized. However, early investigations, constrained by technological limitations, were largely confined to descriptive observations, such as the effects of emotion and stress on immune function.^471–473^ With the rise of molecular biology, specific mechanisms mediating neuro-immune interactions in distinct disease contexts have begun to emerge. For instance, sympathetic nerves regulate ADRB1⁺CD8⁺ T cells in tumors, while ADRB2⁺ macrophages contribute to the immune response in pneumonia.^18,474^ Concurrently, accumulating evidence has revealed that immune dysregulation, particularly inflammatory processes, plays an active role in neurological disorders, exemplified by the involvement of IL-6 in depression.^475^ However, most studies have traditionally portrayed the nervous system as a passive participant in disease rather than as an active hub that senses, integrates, and regulates immune activity. Recent advances have reshaped this perspective. Emerging findings suggest that neuro-immune interactions are rooted in an evolutionary strategy designed to coordinate systemic defense against threats, underscoring the functional parallels between these two systems in terms of signal recognition, information storage/recall, and adaptive response generation. For example, the amygdala has been implicated in antigen-avoidance behavior, whereas the InsCtx acts as a central repository for inflammatory memory.^85,342^ These discoveries mark a conceptual shift in neuroimmunology toward a more integrative framework that views neural and immune functions as dynamically coupled rather than hierarchically separated. Despite this progress, the field remains nascent. Key questions awaiting exploration include how the brain encodes specific immune states, how neural circuits are remodeled by chronic inflammation, and how to best therapeutically harness these interactions.

Conceptually, most current neuro-immune studies still focus on peripheral or tissue-level interactions. In contrast, the neural reflexes and central integration of immune information remain poorly characterized. Although the molecular and cellular mechanisms underlying immune recognition, response, and memory have been extensively delineated, how the nervous system perceives, encodes, and interprets immune signals is far less understood. The CeA, a region essential for antigen-avoidance behavior, is tightly linked to higher-order processes such as reward and learning. Recent studies have shown that SST⁺ neurons within the CeA selectively encode diverse external stimuli and modulate the activity of dopaminergic neurons in the substantia nigra pars compacta (SNc) and ventral tegmental area (VTA), thereby shaping reward-learning dynamics.^350^ Moreover, the CeA forms functional circuits with autonomic centers, including the NTS and lateral paragigantocellular nucleus (LPGi), enabling the brain to regulate visceral function through both sympathetic and parasympathetic outputs.^340^ These findings collectively raise the intriguing possibility that the nervous system harbors an immune center that not only stores immune-related memories but also integrates immune information with visceral activity and higher cognitive functions via telencephalic-autonomic connections. However, how neuronal networks encode immune signals and how the nervous and immune systems form specialized but complementary operational frameworks remain key questions for future investigation. Furthermore, as a global regulatory system, the nervous system processes a wide spectrum of physiological information beyond immunity, including cues from the microbiota, metabolites, and circulating proteins.^476–478^ These factors collectively modulate neuro-immune communication and may determine the adaptive or maladaptive outcomes of the system. Defining how the nervous system bridges immune and non-immune networks will thus be central to advancing the next frontier of neuro-immune research.

Second, building upon the conceptual framework and comparative analysis presented herein, we highlight that neuro-immune interactions exhibit both pronounced heterogeneity and underlying conservation. Heterogeneity is mainly manifested in the tissue-specific and stage-specific differences of neuro-immune interactions. This heterogeneity can explain some of the contradictory findings in cancer neuroscience research, such as the intratumoral sympathetic nerves, which are generally considered to promote tumor growth but exhibit tumor-suppressive effects in pancreatic cancer. To address the above phenomena, we propose a dynamic model based on spatiotemporal heterogeneity to provide an explanatory framework. In this model, the outcomes of neuro-immune regulation are jointly shaped by disease stage, receptor-specific activation, and local decoupling between neurotransmitter sources and their targets. The core assumptions include the following: (1) the effects of the ANS on tumor immunity are dose- and time-dependent, and (2) the ultimate outcome is determined by the disease stage, tissue microenvironment, and receptor subtype expression profile. The spatiotemporal heterogeneity of neuro-immune interactions carries clear physiological implications; for instance, adrenaline-mediated vasodilation and lymphocyte mobilization may act synergistically to facilitate lymphocyte redistribution.^139–141,479,480^ Moreover, current studies typically focus on the influence of a specific type of neuron on a particular immune cell. However, within tissues, multiple neuronal subtypes coexist, and their competitive and cooperative interactions remain largely unexplored. For example, an insightful study revealed the anatomical and functional interplay between the ENS and DRG sensory pathways. In a DSS-induced colitis model, the DRG served as a bridge linking intestinal inflammation with ENS activation-induced aversion.^481^ Based on the important classification features of immune responses, acute and chronic, we propose the conservation of neuro-immune interaction-related diseases. Chronic immune responses encompass a broad spectrum of pathological processes.^482^ We propose that neuro-immune interactions in tumors, immune-related disorders, and chronic infections share fundamental similarities with chronic inflammation, particularly in their characteristic patterns of lymphocyte activation followed by functional exhaustion. Consequently, mechanisms elucidated in diseases characterized by chronic immune responses may provide valuable insights and serve as reference points for other conditions. Neuro-immune interactions in chronic immune responses involve prolonged immune cell activation and tissue remodeling. These responses are often accompanied by sustained macrophage, T-cell, and B-cell activation. For example, sympathetic nerve activity in chronic inflammation may partially attenuate inflammation by releasing NE, which suppresses excessive macrophage activation.^483^ This same anti-inflammatory mechanism can be co-opted by tumors to form an immunosuppressive microenvironment.^484^ To address the heterogeneity and conservation of neuro-immune interactions, comparative analyses across diseases and comprehensive mapping of neuro-immune interactions across tissues are urgently needed.

### Limitations of current technology

Omics technologies hold considerable promise for the systematic analysis of neuro-immune interactions. For example, a recent study integrated single-cell and spatial omics to delineate the spatial landscape of neural infiltration in pancreatic cancer.^102^ Nevertheless, certain limitations remain. The cell bodies of postganglionic neurons that directly innervate tissues are generally located outside the target organs, in distant ganglia or within intrinsic plexuses.^485^ Consequently, the aforementioned studies have relied on myelin-associated markers such as MPZ and MBP as proxies to infer the distribution of neural components.^486,487^ However, such indirect approaches cannot capture dynamic transcriptional changes or the functional states of neurons involved in neuro-immune interactions. To overcome these limitations and directly profile the neurons involved, combining retrograde tracing with single-cell or spatial omics offers a promising strategy to resolve the molecular features of ganglia that innervate specific tissues. Recent work employing retrograde labeling and sequencing of sympathetic neuronal populations projecting to distinct visceral organs revealed organ-specific modular transcriptional architectures, enabling fine-tuned regulation of organ function.^469,488^ This approach provides a powerful technical avenue for elucidating the tissue-specific roles of the nervous system in immune regulation.

Electrical signaling represents one of the most fundamental physiological characteristics of neurons.^489^ Real-time monitoring of the membrane potential not only reflects neuronal excitability but also opens new windows into how neural activity modulates local immune responses. Combining electrophysiology with optogenetics and calcium imaging allows investigators to track the firing patterns of specific neural circuits in vivo and to link these patterns to immune outcomes, thereby integrating molecular-level omics with functional-level readouts. Integrating membrane potential dynamics with transcriptional profiling could further reveal how neural signals shape the immune microenvironment in a spatiotemporally dependent manner.^444,490^ For instance, recent patch-seq studies in glioma have identified hybrid cells exhibiting selective neuronal electrophysiological activity despite morphologically resembling glial cells, with a correlation to patient prognosis. Integration with single-cell omics further elucidated the functional properties of these hybrid cells. This integrated approach, linking electrophysiology with omics in a complex tissue, establishes a method for future studies aiming to dissect how infiltrating neurons or neuron-like cells influence the immune microenvironment through their electrical activity.^491^

### Future directions

Current neuro-immune research has predominantly focused on interactions at the local tissue level, investigating the spatial co-localization, signaling exchanges, and functional consequences between neurons and immune cells under specific tissue or pathological contexts. The ANS integrates the immune states of multiple organs via preganglionic and postganglionic circuits, while the CNS can perceive and modulate immune signals through the autonomic nerve axis. Consequently, future studies should shift from examining “local interactions” toward exploring “global interactions,” aiming to construct cross-tissue, cross-system models of neuro-immune networks to understand how neural signals shape systemic immune homeostasis and immunopathology. At the local level, spatial transcriptomics and spatial proteomics will serve as essential tools for dissecting the neuro-immune niche. We can uncover neuron-type-specific patterns of immune regulation by systematically characterizing the spatial distribution of different neuronal fibers, including sympathetic, parasympathetic, and sensory neurons, and their surrounding immune, stromal, and vascular cells. In the future, integrating spatial transcriptomic data with electrophysiological activity and metabolic signals may enable multidimensional, systems-level analysis of neuro-immune interactions, revealing their dynamic evolution under both physiological and pathological conditions.

## Conclusion

Interactions between the nervous and immune systems have long captivated scientific interest. Recent advances in neuroimmunology have led to a new conceptual framework for neural-immune interactions organized into three interconnected modes: local neuro-immune interactions, neuro-immune reflexes, and brain processing of immune-derived signals. Technological innovations, such as high-resolution sequencing, have propelled the field into a new era, enabling precise identification of neuronal subpopulations that regulate the immune microenvironment beyond traditional histological limits. However, methodological and conceptual divides between neuroscience and immunology, along with the need for anatomically informed interpretation of single-nucleus transcriptomics, continue to hinder integrated research and underscore the need for cross-level theoretical innovation. Importantly, the plasticity of the neuro-immune interactome represents both a vulnerability and a therapeutic opportunity. Future progress depends on deciphering its disease-specific logic and leveraging interdisciplinary tools that bridge neuroscience, immunology, and beyond to achieve precise modulation of this ancient yet adaptive alliance.
